# The incidence, prevalence, and years lived with disability of forearm fractures: a systematic analysis based on the global burden of disease study 2021

**DOI:** 10.3389/fpubh.2025.1598660

**Published:** 2025-07-10

**Authors:** Xiaobo Fan, Zongyou Yang, Yuan Liu, Zhikun Wei, Chenyang Zhao, Chaojian Pang

**Affiliations:** ^1^Department of Orthopaedic Surgery, The First Hospital of Handan, Handan, China; ^2^Department of Orthopaedic Surgery, The Third Hospital of Hebei Medical University, Shijiazhuang, China

**Keywords:** forearm fractures, global burden of disease, incidence, prevalence, years lived with disability, epidemiology

## Abstract

**Purpose:**

Forearm fractures significantly impact global health and socioeconomic systems. This study examines forearm fracture burden across 204 countries and territories from 1990 to 2021.

**Methods:**

Data from Global Burden of Disease (GBD) 2021 were analyzed, including incidence (new cases occurring each year), prevalence (total existing cases at a given time), years lived with disability (YLDs, measuring time lived with reduced health due to injury), and causes categorized by injury mechanisms. Age-standardized rates (ASRs) with 95% uncertainty intervals (UIs) were calculated to address demographic variability. Trends were stratified by region, age, sex, and injury etiology.

**Results:**

Globally, forearm fracture cases increased by 22.25% (from 26.1 to 31.9 million), while age-standardized incidence rates declined by 16.75% (to 402.35 per 100,000). Prevalence rose by 39.12% (from 4.5 to 6.2 million cases), with YLDs increasing by 42.22% (from 144,166 to 205,031). Regional variations were substantial: Oceania experienced the highest incidence surge (150%), whereas Central Europe saw a 32.17% decline. Sex-specific analysis revealed marked disparities: in the ≥75 age groups, female prevalence rates were approximately double those of males (>800 per 100,000 vs. ~300 per 100,000 for ages 95+). Among the older adult (≥60 years), females showed a steeper increase in YLD rates, reaching 30 per 100,000 in the 95 + age group compared with 15 per 100,000 in males. Falls were the predominant cause, particularly in Central/Eastern Europe (>500 per 100,000).

**Conclusion:**

Despite declining age-standardized rates, the absolute cases of forearm fractures is rising due to population aging and uneven healthcare access. Sex-specific prevention strategies are needed, emphasizing osteoporosis screening for postmenopausal women and workplace safety measures in regions with high mechanical-force injuries. Policymakers should prioritize resources for high-burden regions and implement targeted fall prevention programs.

## Introduction

Forearm fractures involve fractures of the radius and/or ulna bones. These injuries contribute to morbidity and disability related to musculoskeletal disorders across all age groups worldwide. As one of the most common types of fractures, they impose substantial socioeconomic costs through healthcare expenditures and result in long-term disabilities and chronic pain, particularly affecting upper limb function (1–3). With equitable distribution of healthcare resources, forearm fractures can be diagnosed early and managed promptly. Thus, the knowledge of the global forearm fracture epidemiology, including incidence patterns, prevalence distribution, and disability burden, is vital for addressing the needs and ensuring a fair allocation of resources in healthcare.

The literature to date has focused mainly on the epidemiological characteristics of forearm fracture patterns (4–7). Numerous studies have examined these patterns, but each has focused on a single country using different methods and inconsistent data. This lack of standardized global monitoring has created significant knowledge gaps regarding trends, disparities, and the true global burden of forearm fractures. Consequently, a comprehensive global burden assessment that includes fracture trends across countries, age groups, and sexes has remained unexplored. To our knowledge, no systematic global analyses of this scope have been conducted to date.

The Global Burden of Disease (GBD) Study 2021 provides a unique foundation for systematic global epidemiological analysis across 204 countries and territories (8–14). The GBD database provides a unique foundation for calculating the worldwide burden of forearm fractures. Using this data, we calculated incidence (new cases occurring each year), prevalence (total existing cases at a given time), years lived with disability (YLDs, measuring time lived with reduced health due to injury) for 204 countries and territories. The findings can offer help in the development of primary prevention programs that focus on regions and populations with an increased fracture risk. In addition, the study will act as a support for prioritization and resource allocation by calculating the burden of forearm fractures.

## Methods

### Data source

The data used in this analysis were derived from the GBD study from 1990 to 2021, which is maintained by the Institute for Health Metrics and Evaluation at the University of Washington, and accessible at https://vizhub.healthdata.org/gbd-results. GBD 2021 offers a detailed assessment of both all-cause and cause-specific incidence, prevalence, mortality, YLDs, Years of Life Lost (YLLs), and Disability-Adjusted Life Years (DALYs) for 371 diseases and injuries, across 204 countries and territories from 1990 to 2021. The general methodology of the GBD study has been published previously (13, 15, 16). The data were extracted from various sources, such as civil registration records, vital statistics, household surveys, insurance claims data, and hospital reports (17).

The GBD study classifies injuries into two categories: nature of injury and cause of injury. Nature of injury refers to the physical trauma that occurs in the patient’s body, while cause of injury denotes the mechanism of these injuries, such as falls or road injuries. Forearm fractures are categorized as a nature of injury rather than a cause of injury. Consequently, only the nonfatal burden (YLDs) for forearm fractures was evaluated, rather than YLLs. In the GBD 2021 study, causes of injuries are organized from Level 1 to Level 4. The Level 1 cause is “Injuries,” with three Level 2 causes: Transport injuries, Unintentional injuries, and Self-harm and interpersonal violence. Transport injuries comprise two Level 3 causes: road injuries and other transport injuries. Unintentional injuries include 11 Level 3 causes: falls, drowning, fire, heat and hot substances, poisonings, exposure to mechanical forces, medical treatment side effects, animal contact, foreign bodies, environmental heat and cold exposure, and exposure to forces of nature. Self-harm and interpersonal violence include four Level 3 causes: self-harm, interpersonal violence, conflict and terrorism, and police conflict and executions.

Forearm fractures are defined as fractures of radius and/or ulna. According to the International Classification of Diseases, Tenth Edition (ICD-10), these injuries are classified under code S52, which includes fractures of the upper end of the ulna (S52.0), upper end of the radius (S52.1), shaft of the ulna (S52.2), shaft of the radius (S52.3), shafts of both ulna and radius (S52.4), lower end of the radius (S52.5), lower end of both ulna and radius (S52.6), multiple fractures of the forearm (S52.7), other parts of the forearm (S52.8), and unspecified parts of the forearm (S52.9).

The calculation of age-standardized rates (ASRs) in the GBD Study 2021 utilized a direct method of standardization. The reference population (a standard population age structure that serves as a common baseline) used for standardization has been described in detail in previous publications (18). In our study, ASRs for forearm fractures were directly obtained from the GBD 2021 Results Tool interface,^1^ where the option “Age-standardized” is explicitly available.

This study classified people under 5 years of age and over 95 years of age each into single groups. Given this, the age groups were defined as 0–4, 5–9, 10–14, 95+. To extract the relevant data from the GBD database, the search parameters were set as follows: 1. GBD estimate - ‘Injuries by nature’, 2. Measure - ‘Incidence’, ‘prevalence’, and ‘YLDs’, 3. Metric - ‘Numbers’, ‘Percent’, ‘rate’, 4. Injury - ‘Fracture of radius and/or ulna’, 5. Cause - ‘All cause’ and Level 3 causes, 6. Location - ‘Global’, ‘all GBD regions’, and ‘all countries and territories’, 7. Age - ‘All ages’, ‘Age-standardized’, and all the defined age groups, 8. Sex - ‘Male’, ‘Female’, and ‘Both’. All the target data and the total percentage change data were searched and downloaded ranging from 1990 to 2021. This study used publicly available data, which do not contain any individual patient information. Therefore, ethical approval was not required.

### Statistical analyses

The number and rates of forearm fractures per 100,000 population with the corresponding 95% uncertainty intervals (UIs) were used. Uncertainty in the GBD study is produced via multiple mechanisms, such as sampling errors, methodological means for data adjustment and standardization, uncertainties regarding the model-fitting coefficients, the variability of severity gradings and disability weights. To quantify this uncertainty, we used the Bayesian Meta-Regression method that provides posterior distributions of each parameter estimate. To incorporate this uncertainty in our analysis, we obtained 1,000 samples from the posterior distributions of the incidence, prevalence, and YLDs due to forearm fractures to evaluate the final 95% UIs. The final estimate was achieved following 1,000 iterations of the sample model. The uncertainty intervals of 95% were determined by the 2.5th and 97.5th percentiles of those samples. The 95% UIs should be considered as Bayesian credible intervals, which indicate the range that is assumed to contain the true parameter (value) of interest with a 95% probability based on the posterior distribution. When comparing between regions or countries, non-overlapping UIs indicate statistically meaningful differences. Overlapping intervals suggest that observed differences may reflect modeling uncertainty. The width of UIs varies by data availability, with regions having limited input data showing wider intervals and lower confidence in precise estimates. We were able to estimate the burden of forearm fractures globally, across all individual countries, and within the 21 GBD-defined regions, including Western Europe, Eastern Europe, Central Europe, South Asia, Southeast Asia, East Asia, Central Asia, High-income North America, High-income Asia Pacific, Australasia, North Africa and the Middle East, the Caribbean, Latin America (Andean, Central, Tropical, Southern), Oceania, and Sub-Saharan Africa (Western, Central, Eastern, Southern). These regional classifications were published earlier (8, 9, 15, 19–22). YLDs, incidence and prevalence of forearm fractures across sex and age group were reported. Given the variations in age distribution in the GBD 2021 dataset, it is essential to adjust for differences in age structure. The age-standardized rates represent disease rates calculated per 100,000 population after adjusting for differences in age distribution. Therefore, we used the age-standardized rate (ASR, per 100,000 individuals) to ensure the comparability. Age-standardized rates were obtained from the GBD 2021 database (see text footnote 1, respectively). Percentage change in ASRs represents the relative change in age-standardized rates between 1990 and 2021, calculated as [(ASR₂₀₂₁ - ASR₁₉₉₀)/ASR₁₉₉₀] × 100%. A world map was generated, consisting of 204 countries and territories, in order to visualize the burden of fractures in 2021. R software version 4.4.2 (R Foundation for Statistical Computing, Vienna, Austria) was used to perform statistical analyses and create all figures and tables. The R script used for figure and table generation is included in Supplementary File 1. The statistical significance levels were set at *p* < 0.05.

## Results

### Incidence of forearm fractures

The global cases of forearm fractures from 1990 to 2021 increased by 22.25%, from 26,098,810 (95% UI: 20,967,988-32,372,267) to 31,905,396 (95% UI: 25,403,829-39,982,115), with vary in regional disparities. East Asia had the largest number of cases and substantial increase of cases (+30.34%) among all the regions, increasing from 4,527,959 (95% UI: 3,647,766-5,653,521) in 1990 to 5,901,599 (95% UI: 4,594,522-7,549,669) in 2021. The rise of incidence cases of Oceania was the most prominent compared to other regions [from 19,396 (95% UI: 15,756-24,078) to 48,970 (95% UI: 39,493-61,705)]. On the other hand, a less constant trend was observed for Western Europe and Central Asia, with a small increase of 1.23 and 2.29%, respectively. A decreasing trend was observed in several places including Central Europe (−32.17%) as well as High-income Asia Pacific, Central Latin America, and Eastern Europe (Table 1).

**Table 1:** Incident cases, prevalent cases, and years lived with disability (YLDs) numbers due to forearm fractures in patients from 1990 to 2021.

Region	Incidence cases			Prevalence cases			YLDs numbers		
	Year 1990 (95% UI)	Year 2021 (95% UI)	Overall change (%)	Number in 1990 (per 10,000, 95% UI)	Year 1990 (95% UI)	Year 2021 (95% UI)	Overall change (%)	Number in 2021 (per 10,000, 95% UI)	Year 1990 (95% UI)
Global	26,098,810 (20,967,988,32,372,267)	31,905,396 (25,403,829,39,982,115)	22.25	4,452,829 (3,572,654,5,448,206)	6,194,792 (5,084,822,7,507,976)	**39.12**	144,166 (87,129,229,017)	205,031 (126,061,320,235)	**42.22**
High-income Asia Pacific	730,922 (546,225,957,520)	559,474 (411,483,742,966)	−23.46	138,458 (109,907,172,206)	139,886 (115,803,166,598)	1.03	4,614 (2,807,7,390)	4,836 (3,074,7,479)	4.82
High-income North America	1,031,139 (773,113,1,344,019)	1,336,445 (998,791,1,792,531)	29.61	212,340 (172,945,259,809)	327,222 (273,423,394,328)	**54.10**	7,116 (4,405,11,234)	11,101 (6,943,17,210)	**56.00**
Western Europe	1,965,226 (1,415,075,2,630,037)	1,989,413 (1,401,961,2,695,634)	1.23	416,112 (333,364,511,198)	480,404 (391,669,584,283)	15.45	14,002 (8,593,21,744)	16,412 (10,265,25,127)	17.21
Australasia	121,939 (89,712,157,537)	174,378 (128,593,231,022)	**43.00**	22,311 (17,596,28,077)	36,600 (29,363,44,898)	**64.05**	732 (444,1,163)	1,222 (743,1898)	**66.90**
Andean Latin America	174,473 (145,048,209,115)	268,722 (216,275,327,400)	**54.02**	26,048 (20,673,32,626)	45,210 (36,347,55,161)	**73.56**	817 (485,1,341)	1,463 (877,2,324)	**78.99**
Tropical Latin America	1,246,155 (985,576,1,578,313)	1,323,439 (1,052,472,1,654,671)	6.20	193,924 (150,733,243,289)	246,242 (201,987,297,428)	26.98	6,138 (3,626,9,642)	8,088 (4,862,12,531)	**31.77**
Central Latin America	1,285,069 (1,016,928,1,602,942)	1,249,753 (995,244,1,539,091)	−2.75	194,536 (151,140,247,194)	222,214 (179,604,272,383)	14.23	6,120 (3,597,10,050)	7,258 (4,378,11,524)	18.60
Southern Latin America	177,637 (134,263,227,368)	240,010 (179,411,308,000)	**35.11**	30,659 (23,883,38,899)	45,671 (36,390,56,653)	**48.96**	996 (601,1,585)	1,511 (914,2,394)	**51.71**
Caribbean	138,314 (115,038,164,870)	205,406 (169,932,246,153)	**48.51**	22,935 (18,629,28,133)	39,100 (32,749,46,640)	**70.48**	739 (444,1,208)	1,286 (807,2008)	**74.09**
Central Europe	1,453,791 (1,170,269,1,763,969)	986,092 (785,348,1,214,142)	**−32.17**	263,556 (215,708,322,480)	208,854 (174,783,251,121)	−20.76	8,611 (5,232,13,746)	7,008 (4,328,10,975)	−18.61
Eastern Europe	2,597,606 (2,129,496,3,149,138)	1,830,109 (1,476,289,2,262,577)	−29.55	477,557 (394,352,579,600)	375,461 (314,910,450,901)	−21.38	15,724 (9,458,25,074)	12,589 (7,674,19,662)	−19.94
Central Asia	485,675 (395,740,588,023)	496,808 (403,576,601,441)	2.29	74,584 (59,343,92,812)	82,077 (66,250,101,275)	10.05	2,362 (1,408,3,800)	2,648 (1,603,4,176)	12.08
North Africa and Middle East	2,000,794 (1,662,872,2,376,440)	3,093,154 (2,534,054,3,770,699)	**54.6**	299,377 (236,621,369,676)	507,995 (411,487,627,560)	**69.68**	9,391 (5,562,15,249)	16,298 (9,855,26,127)	**73.55**
South Asia	4,918,976 (3,937,728,6,230,424)	7,486,598 (5,984,746,9,493,063)	**52.20**	778,743 (609,851,984,744)	1,358,856 (1,102,829,1,671,677)	**74.49**	24,624 (14,474,39,226)	44,122 (26,815,67,448)	**79.18**
Southeast Asia	1,758,877 (1,459,674,2,089,414)	2,158,505 (1,756,110,2,637,723)	22.72	278,446 (223,640,337,695)	394,605 (324,351,475,429)	**41.72**	8,871 (5,242,14,291)	12,986 (8,010,20,416)	**46.38**
East Asia	4,527,959 (3,647,766,5,653,521)	5,901,599 (4,594,522,7,549,669)	**30.34**	801,910 (651,353,990,183)	1,279,923 (1,074,175,1,535,524)	**59.61**	26,379 (15,830,41,353)	43,389 (27,324,67,042)	**64.48**
Oceania	19,396 (15,756,24,078)	48,970 (39,493,61,705)	**152.48**	3,053 (2,406,3,815)	8,085 (6,486,10,091)	**164.82**	97 (58,155)	260 (155,406)	**167.17**
Western Sub-Saharan Africa	482,448 (396,750,582,283)	1,105,118 (907,977,1,353,067)	**129.06**	72,824 (57,767,90,193)	167,455 (132,635,207,770)	**129.95**	2,288 (1,354,3,733)	5,276 (3,081,8,525)	**130.61**
Eastern Sub-Saharan Africa	655,378 (512,060,922,409)	938,523 (777,063,1,144,677)	**43.20**	92,604 (70,897,125,454)	143,690 (114,020,177,881)	**55.17**	2,852 (1,629,4,883)	4,534 (2,690,7,343)	**59.00**
Central Sub-Saharan Africa	144,757 (120,548,175,757)	308,510 (256,018,376,675)	**113.12**	21,970 (17,443,26,884)	48,334 (38,720,59,086)	**120.00**	692 (409,1,164)	1,536 (916,2,491)	**122.04**
Southern Sub-Saharan Africa	182,278 (153,615,215,465)	204,372 (172,550,244,390)	12.12	30,884 (25,514,37,469)	36,908 (30,614,44,571)	19.50	1,001 (605,1,605)	1,210 (728,1914)	20.84

Overall change represents the relative change between 1990 and 2021. The 95% UIs (uncertainty intervals) represent the range within which there is a 95% probability that the true value lies. Values in bold indicate regions experiencing substantial changes (>30%) during the study period.

**Figure 1:**
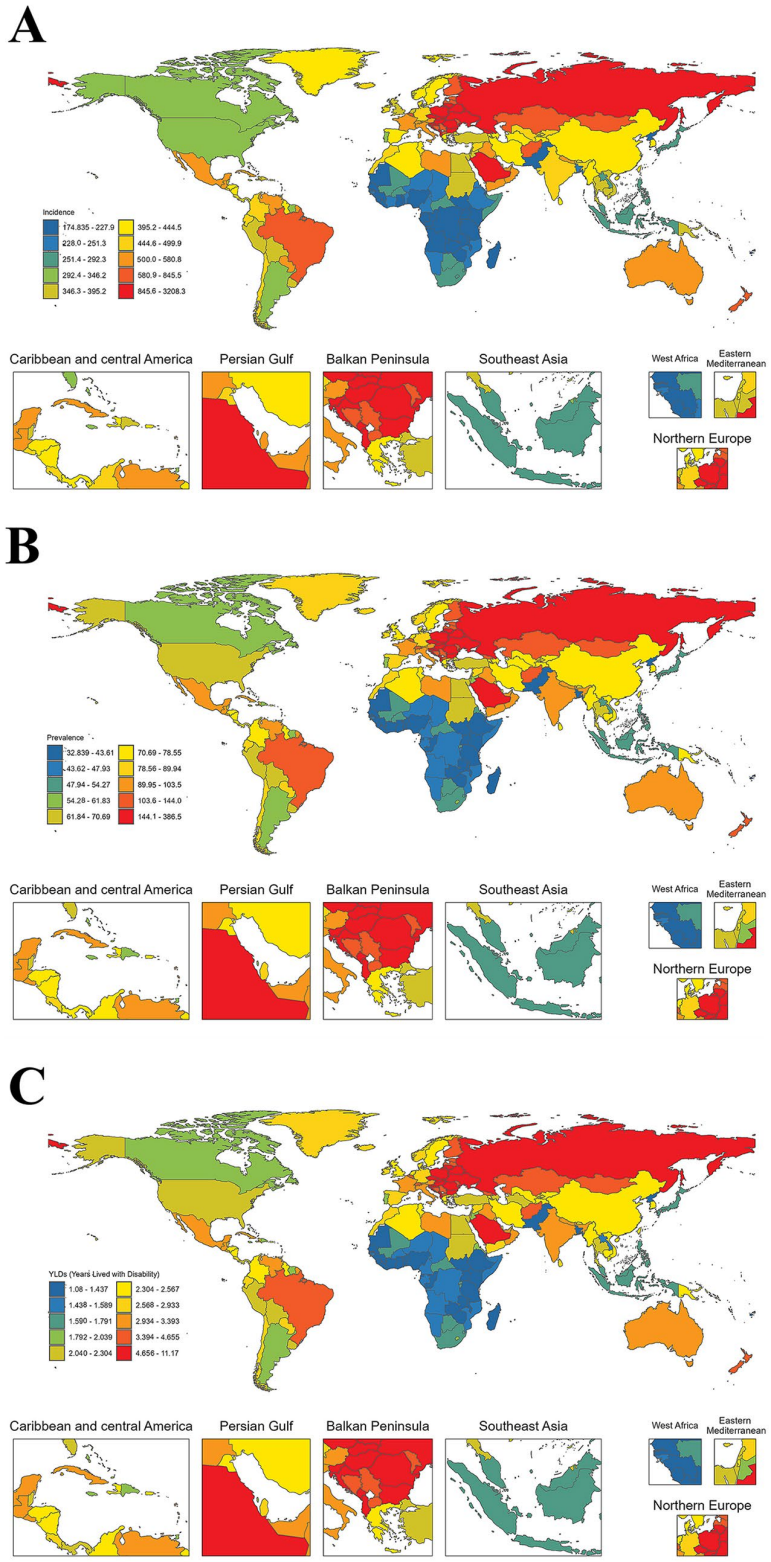
The global burden of forearm fractures in 204 countries and territories, 2021. Countries are color-coded using a progressive scale where dark blue represents the lowest burden, progressing through green and yellow to dark red representing the highest burden. Insets provide detailed views for the Caribbean and Central America, Persian Gulf, Balkan Peninsula, Southeast Asia, West Africa, Eastern Mediterranean, and Northern Europe regions. **(A)** The age-standardized incidence rate (per 100,000 population). **(B)** The age-standardized prevalence rate (per 100,000 population). **(C)** The age-standardized YLDs rate (per 100,000 population).

Although the age-standardized incidence rate has continuously risen globally, the global age-standardized incidence rate decreased by 16.75% to 402.35 per 100,000 (95% UI: 319.86–505.21) in 2021. The highest incidence rate of this proportion of population has been in Australasia with 943.29 per 100,000 (95% UI: 755.11–1173.17), followed by Central Europe with 909.72 per 100,000 (95% UI: 720.21–1119.30). There was a substantial decline of rates as well (−30.64% and −30.62%, respectively, in Central Latin America and Eastern Sub-Saharan Africa; as shown in Table 2). The magnitude of the geographic inequalities remained large with great divides observed across 204 nations and territories (Figure 1A). Slovenia maintained the highest level of age-standardized rates ASR (per 100,000) from 1990 to 2021: 1,171.1-1,831.2 in ASR in 1990 and 916.7–1,469.7 in ASR in 2021. Kiribati consistently recorded the lowest ASR. Bhutan showed the greatest ASR increase (+37.3%) between 1990 and 2021, while Congo had the highest negative percentage change (−67.3) in the same period (Supplementary Table 1).

**Table 2:** Incident, prevalent, and years lived with disability (YLDs) rate due to forearm fractures in patients from 1990 to 2021 (per 100,000 population).

Region	Incidence		Prevalence		YLDs	
	Year 2021 (95% UI)	Change (%)	Year 2021 (95% UI)	Change (%)	Year 2021 (95% UI)	Change (%)
Global	402.35 (319.86,505.21)	−16.75	76.22 (62.45,92.62)	−15.66	2.51 (1.54,3.93)	−15.69
High-income Asia Pacific	313.44 (228.19,416.96)	−26.74	56.42 (43.39,71.46)	−26.56	1.86 (1.13,3)	−26.57
High-income North America	306.6 (228.67,402.31)	−15.32	63.33 (51.73,77.46)	−8.96	2.1 (1.29,3.33)	−8.71
Western Europe	458.91 (323.65,631.21)	−11.01	84.18 (64.26,105.51)	−12.2	2.76 (1.65,4.41)	−12.39
Australasia	594.58 (428.55,792.88)	−4.83	103.55 (79.19,129.77)	−4.89	3.35 (1.98,5.37)	−5.11
Andean Latin America	399.69 (321.22,485.96)	−7.08	68.92 (55.66,83.84)	−5.92	2.24 (1.35,3.55)	−5.64
Tropical Latin America	585.42 (462.38,738.52)	−23.93	104.16 (83.92,126.71)	−23.03	3.39 (2.03,5.25)	−22.96
Central Latin America	499.4 (396.56,617.5)	**−30.64**	88.07 (71.14,108.15)	**−30.85**	2.87 (1.73,4.56)	**−30.9**
Southern Latin America	366.66 (272.14,471.41)	3.34	64.27 (50.18,80.53)	2.17	2.09 (1.25,3.35)	1.84
Caribbean	436.1 (362.5,523.7)	13.88	79.21 (65.9,95.07)	13.87	2.58 (1.61,4.06)	13.16
Central Europe	909.72 (720.21,1119.3)	−22.81	154.7 (124.07,192.46)	−24.48	4.99 (2.99,8.05)	−24.67
Eastern Europe	943.29 (755.11,1173.17)	−19.81	162.13 (130.19,200.07)	−20.08	5.25 (3.12,8.3)	−20.25
Central Asia	515.48 (417.89,624.25)	−21.34	87.19 (70.45,107.33)	−21.13	2.82 (1.71,4.44)	−21.27
North Africa and Middle East	486.29 (399.4,591.02)	−11	84.72 (69.1,104.13)	−10.72	2.75 (1.67,4.37)	−11
South Asia	412.64 (327.21,524.1)	−10.95	82.21 (67.94,100.74)	−6.96	2.7 (1.67,4.1)	−6.41
Southeast Asia	310.83 (253.13,380.06)	−16.01	58.39 (48.06,70.2)	−14.8	1.92 (1.19,3.02)	−14.67
East Asia	397.63 (312.39,507.23)	7.91	75.85 (61.85,92.99)	4.84	2.51 (1.53,3.93)	3.6
Oceania	357.76 (287.63,449.57)	20.67	69.79 (57.99,85.17)	21.2	2.31 (1.41,3.56)	20.76
Western Sub-Saharan Africa	225.5 (186.58,272.76)	−5.48	42.89 (35.55,51.41)	−3.72	1.42 (0.86,2.21)	−3.25
Eastern Sub-Saharan Africa	221.73 (185.08,265.61)	**−30.62**	42.54 (35.28,50.94)	−22.78	1.4 (0.87,2.18)	−21.05
Central Sub-Saharan Africa	225.4 (187.62,270.76)	−9.37	44.56 (37.2,52.31)	−6.01	1.48 (0.91,2.33)	−5.37
Southern Sub-Saharan Africa	247.45 (209.04,294.67)	−27.42	48.52 (40.9,57.84)	−29.62	1.61 (0.98,2.54)	**−30.41**

Overall change represents the relative change between 1990 and 2021. The 95% UIs (uncertainty intervals) represent the range within which there is a 95% probability that the true value lies. Values in bold indicate regions experiencing substantial changes (>30%) during the study period.

**Figure 2:**
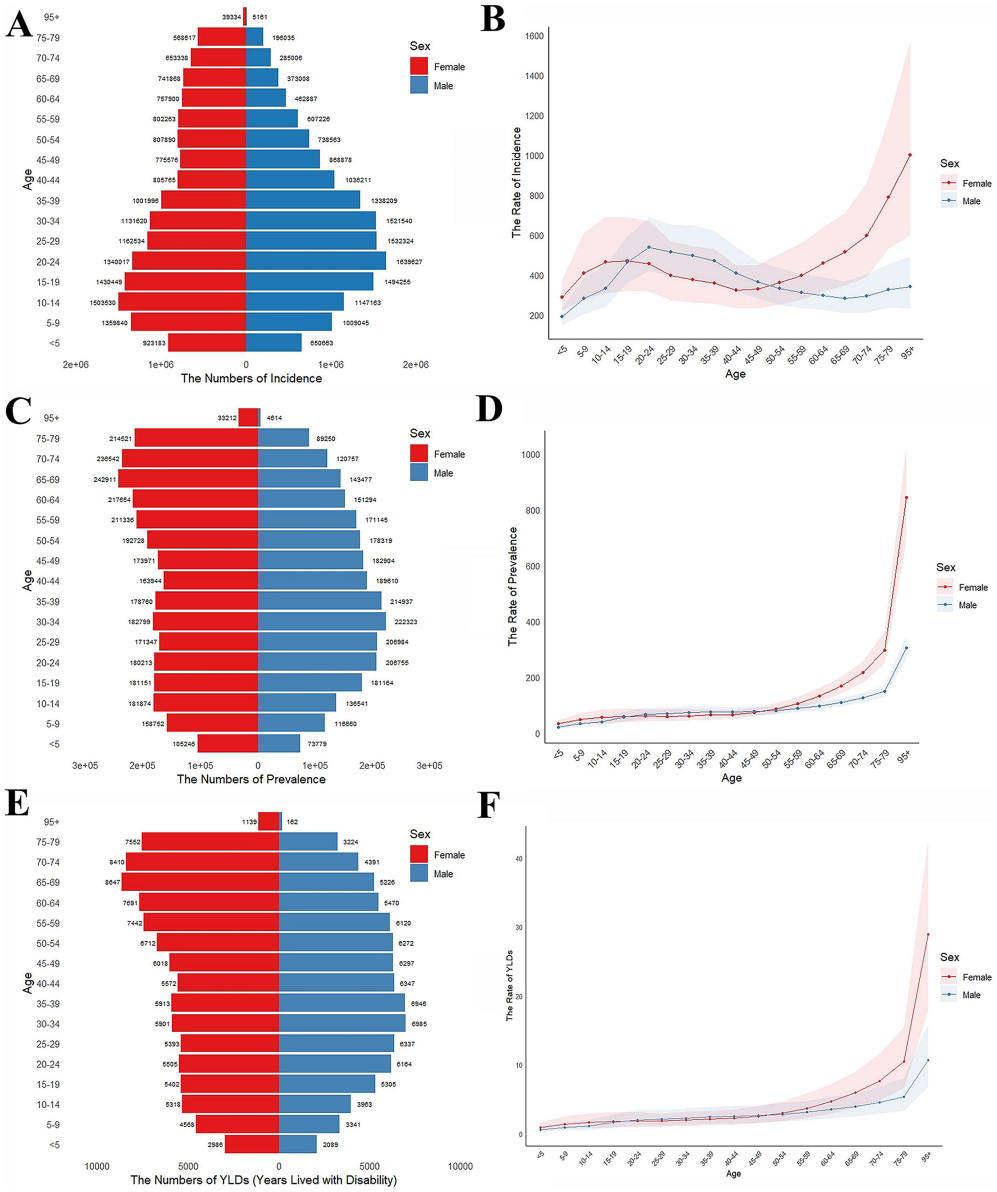
Number and age-standardized incidence, prevalence, and YLDs rates of forearm fractures by age group and sex in 2021. **(A)** Numbers of incidence by age group and sex. **(B)** Age-standardized rates of incidence per 100,000 population by age group and sex; shaded areas indicate 95% UIs. **(C)** Numbers of prevalence by age group and sex. **(D)** Age-standardized rates of prevalence per 100,000 population by age group and sex; shaded areas indicate 95% UIs. **(E)** Numbers of YLDs by age group and sex. **(F)** Age-standardized rates of YLDs per 100,000 population by age group and sex; shaded areas indicate 95% UIs.

**Figure 3:**
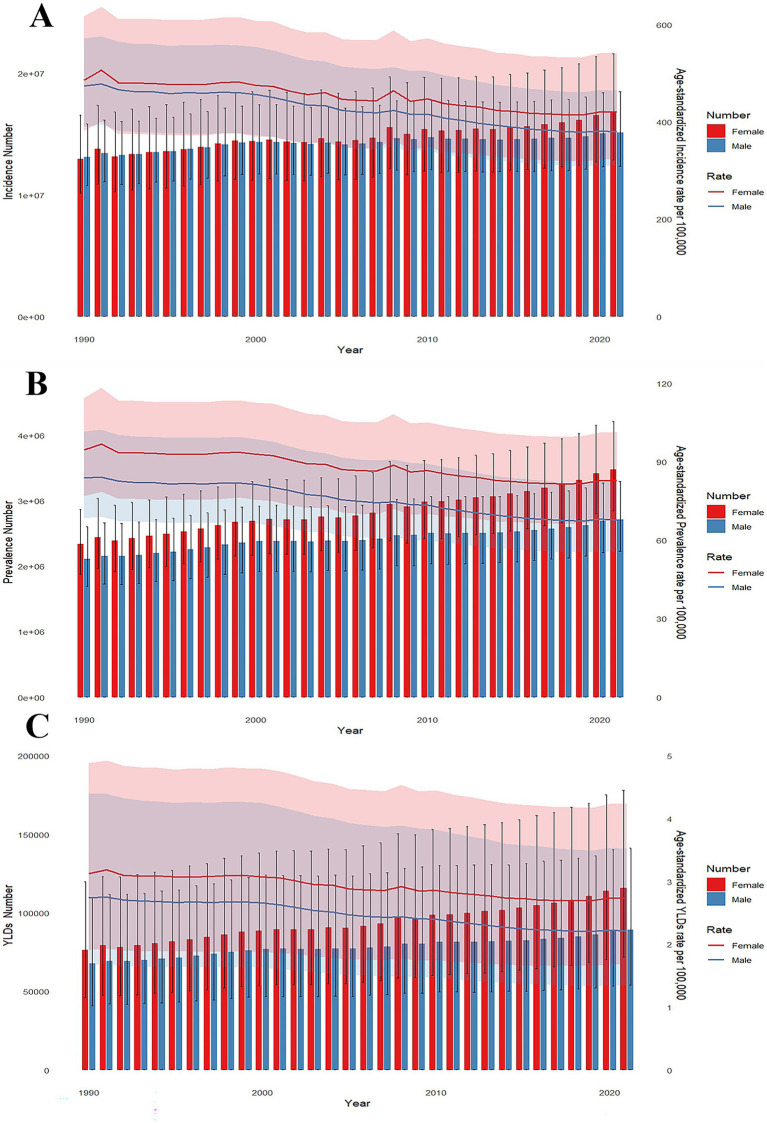
Trends in the all-age cases and age-standardized incidence, prevalence, and YLDs rates of forearm fractures by sex from 1990 to 2021. Bars represent total prevalence numbers with error bars indicating 95% UIs; lines and shaded areas indicate age-standardized rates and their corresponding 95% UIs, respectively. **(A)** Trends in incidence number and age-standardized incidence rates per 100,000 population. **(B)** Trends in prevalence number and age-standardized prevalence rates per 100,000 population. **(C)** Trends in YLDs number and age-standardized YLDs rates per 100,000 population.

Sex-specific age distribution patterns are complex. Teenage females (10–14 years) and young males (20–24 years) showed peak incidence rates with 1,503,530 females and 1,639,927 males. After age 40, male incidence case numbers gradually declined while female incidence case numbers remained stable (Figure 2A). Both male and female incidence rates were increasing at a stable rate before early adulthood (<20 years), followed by a period of decline. After this decline, females resumed an upward trend, reaching approximately 1,000 per 100,000 in the 95 + age group. In contrast, males continued to decline, with only a slight upward shift in the oldest age groups (Figure 2B). The uncertainty intervals are notably wider for females at younger and older ages, reflecting lower absolute case numbers in pediatric populations and smaller population sizes in the oldest age groups. There has been an upward trend for both female and male incidence case numbers from 1990 to 2021. However, while the age-standardized incidence rates continued to drop, the rates for females remained consistently higher than that for males (Figure 3A).

### Prevalence of forearm fractures

Between 1990 and 2021, global cases of prevalent forearm fractures rose by 39.12%, from 4,452,829 (95% UI: 3,572,654-5,448,206) to 6,194,792 (95% UI: 5,084,822-7,507,976). At the GBD regional level, Oceania experienced the highest increase (164.82%), followed by Western Sub-Saharan Africa (129.95%) and Central Sub-Saharan Africa (120.00%). Only Central Europe (−20.76%) and Eastern Europe (−21.38%) showed declines (Table 1).

The age-standardized prevalence rate (ASPR) declined by 15.66% globally, reaching 76.22 per 100,000 population (95% UI: 62.45–92.62) in 2021. Eastern Europe reported the highest rate at 162.13 per 100,000 (95% UI: 130.19–200.07), followed by Central Europe (154.70 per 100,000; 95% UI: 124.07–192.46). Sub-Saharan African regions exhibited the lowest rates, with Western Sub-Saharan Africa at 42.89 per 100,000 (95% UI: 35.55–51.41) and Eastern Sub-Saharan Africa at 42.54 per 100,000 (95% UI: 35.28–50.94) (Table 2). At the country level, Russia, shown in red in Figure 1B, had the highest prevalence rates (144.0–386.5 per 100,000), along with several Eastern European countries, such as Ukraine and Belarus. China and India have moderate to high rates (70.69–103.5 per 100,000), while the US shows higher rates than Canada. African nations showed the lowest rates (32.84–43.61 per 100,000) (Figure 1B).

Forearm fracture prevalence showed distinct age- and sex-specific patterns. Among males, case numbers peaked in the young age group (30–34 years), with 222,323 cases. Females exhibited bimodal peaks: the first in the 10–14-year group (181,174 cases) and the second in the 65–69-year group (242,911 cases) (Figure 2C). Prevalence rates in both sexes remained stable and equal for those under 50 years of age (50–100 per 100,000). Thereafter, rates increased sharply, especially among females. By ages 75–79 years, the prevalence rate in females was nearly double that of males. The disparity widened further in the 95 + age group, with female rates exceeding 800 per 100,000 compared to males at around 300 per 100,000 (Figure 2D).

From 1990 to 2021, prevalence case numbers increased for both sexes, with females consistently outnumbering males. Female cases rose from approximately 2.5 million to 3.5 million, while male cases increased from about 2.0 million to 2.8 million. The ASPRs declined globally for both sexes, with females maintaining higher rates throughout the study period (Figure 3B).

### YLDs of forearm fractures

Globally, forearm fracture disability burden (YLDs) increased by 42.22% between 1990 and 2021, from 144,166 (95% UI: 87,129-229,017) to 205,031 (95% UI: 126,061–320,235). Regionally, Oceania (+167.17%), Central Sub-Saharan Africa (+122.04%), and South Asia (+79.18%) experienced the highest increases, while Central Europe (−18.61%) and Eastern Europe (−19.94%) showed reductions (Table 1). Country-level YLD variations are detailed in Supplementary Table 2. China recorded the highest absolute YLD burden for forearm fractures [25,546 (95% UI: 15,320-40,100) in 1990; 42,524 (95% UI: 26,780-65,689) in 2021], while Slovenia maintained the highest age-standardized rates [8.5 (95% UI: 5.2–13.5) per 100,000 in 1990; 6.6 (95% UI: 4.0–10.5) in 2021]. Saudi Arabia showed a 32.6% increase (95% UI: 20.2–45.7%), while Djibouti experienced a 49.1% decrease (95% UI: −67.3% to −27.8%; Figure 1C).

The global YLDs rate per 100,000 population declined by 15.69%, from 2.98 (95% UI: 1.8–4.7) to 2.51 (95% UI: 1.54–3.93) during the period between 1990 and 2021. In 2021, Eastern Europe recorded the highest YLDs rate (5.25 per 100,000; 95% UI: 3.12–8.30), followed closely by Central Europe (4.99 per 100,000; 95% UI: 2.99–8.05). In contrast, Eastern Sub-Saharan Africa reported the lowest rate (1.40 per 100,000; 95% UI: 0.87–2.18). Oceania saw an increase of 20.76% in YLD rates, while Saudi Arabia experienced the greatest decrease of 30.41% between 1990 and 2021 (Table 2).

YLDs distribution showed an evident divergence across the sexes. In females, YLDs rose consistently and peaking around age of 10 years. While male YLDs remained more consistent throughout childhood. Both sexes exhibited elevated YLDs after age 60 (Figure 2E). Age-standardized YLDs increased consistently with age for both males and females. By age 95 and above, female rates peaked at 30 per 100,000, double the male rate (Figure 2F). Similar to trends in prevalence and incidence, YLDs numbers rose steadily for both sexes from 1990 to 2021, whereas age-standardized rates declined. Females exhibited persistently higher absolute YLD numbers and age-standardized YLDs rates for forearm fractures compared to males throughout this period (Figure 3C).

### Global causes of forearm fractures

**Figure 4:**
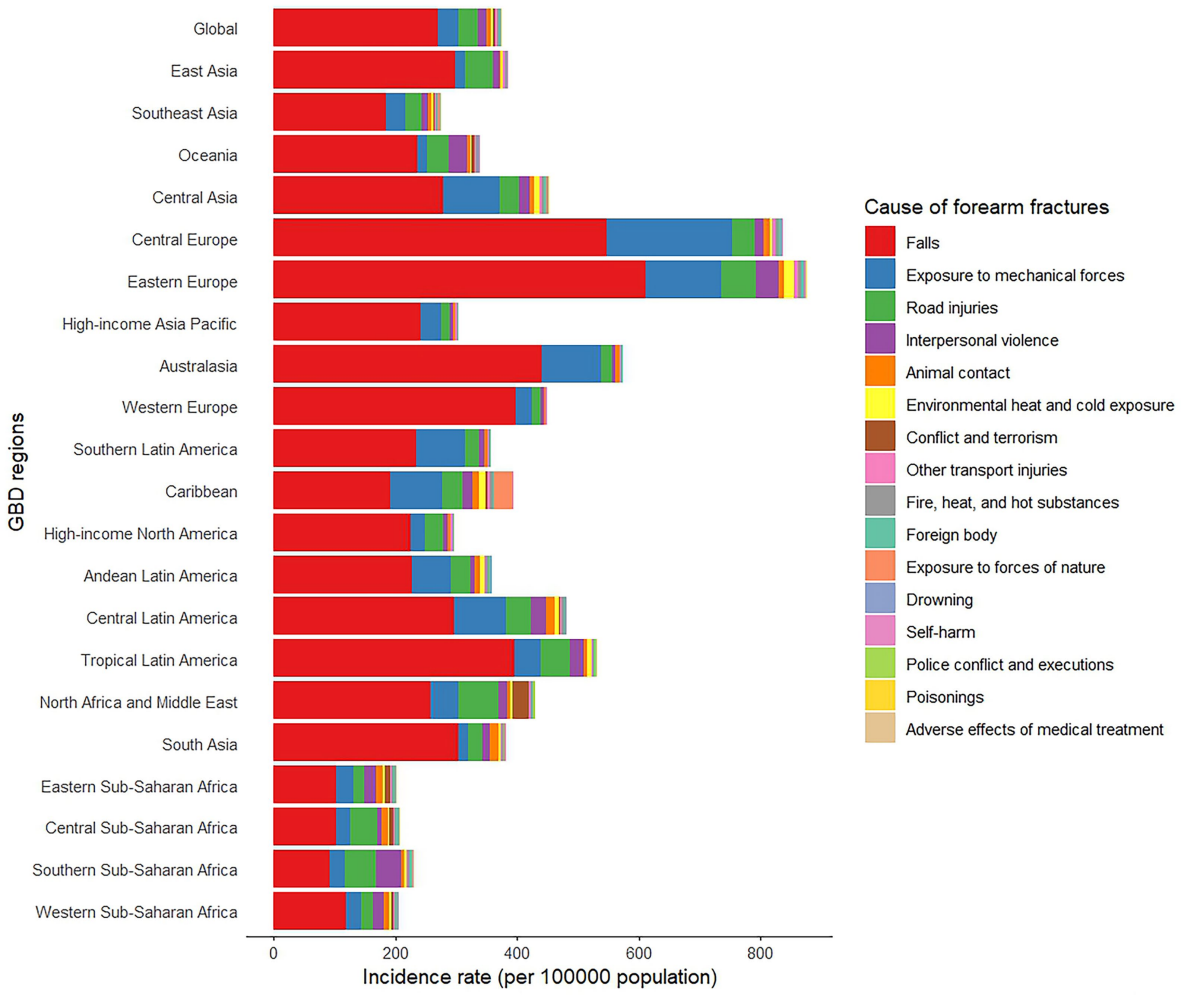
Causes of forearm fractures for the age-standardized incidence rate across GBD regions in 2021. Horizontal stacked bars illustrate incidence rates (per 100,000 population) categorized by causes of fractures, including falls, mechanical forces, road injuries, interpersonal violence, animal contact, environmental exposures, and other external factors. Each color represents a different injury cause.

Falls were the main global cause of forearm fractures and accounted for the highest portion of incidence rates in nearly all regions, with Central and Eastern Europe seeing the highest rates (over 500 per 100,000). The second most significant contributor was exposure to mechanical forces, particularly prominent in Central and Eastern Europe, indicating industrial or workplace-related hazards. Road injuries also contributed substantially in some regions: Southeastern Europe, North Africa, the Middle East, and Southern Sub-Saharan Africa, indicating transportation-related risks. Interpersonal violence, conflict, and terrorism contributed to small proportions worldwide and slightly appeared across Oceania, Southern Sub-Saharan Africa, and Eastern Europe, indicating local security challenges. Animal contact-related injuries were more common in the Caribbean than in other GBD regions. Less common causes, such as self-harm, and environmental exposures, had a slight impact (Figure 4).

## Discussion

This study provides the first comprehensive analysis of global forearm fracture burden using GBD data from 1990 to 2021. Our findings reveal distinct epidemiological patterns across regions, age groups, and sexes. These results highlight the substantial health burden imposed by forearm fractures and provide evidence for targeted prevention strategies and resource allocation.

The GBD Study 2021 employs DisMod-MR 2.1, a sophisticated Bayesian meta-regression tool that integrates data from multiple sources worldwide, enabling standardized comparisons across 204 countries and territories (23). In regions with abundant high-quality data, estimates primarily reflect observed patterns. Conversely, for areas with limited local data, the model leverages epidemiological principles and information from similar regions or comparable time periods to generate informed estimates, thereby ensuring comprehensive global coverage. Additionally, the framework maintains internal epidemiological consistency between incidence, prevalence, and YLDs. Our methodological approach aligns with that of Wu et al. (15). Bayesian meta-regression was employed to estimate incidence, prevalence, and YLDs, using 1,000 iterations to determine uncertainty intervals.

Our findings show that the global incidence of forearm fractures was more than 22% higher than it was in 1990. This trend is similar to those reported in previous studies focusing on other injuries. Even though the number of global forearm fractures went up, the age-standardized incidence rate has declined by 16.75% since 1990, suggesting improvements in regional healthcare access and other preventive measures. This finding is consistent with other GBD analyses, where total numbers rise alongside aging populations despite stable or declining age-standardized rates. Several authors reported a decline in age-standardized rates of forearm and other fractures across many parts of Europe and North America, likely attributable to improvements in preventive healthcare, fall prevention programs, and better management of osteoporosis (24–26). Central Europe’s 22.81% decline in incidence rate aligns with its robust workplace safety regulations and osteoporosis management programs, as reported in previous regional studies (27, 28). Interestingly, our study found that the incidence rate in Oceania remained high, likely driven by a combination of higher rates of falls among the older adult population and trauma related to outdoor activities.

The global patterns we observed reveal complex interplay between demographic and healthcare factors influencing forearm fracture burden. The trend of increasing absolute numbers highlights the profound impact of population aging on musculoskeletal injury burden worldwide. This phenomenon creates unique challenges for healthcare systems, particularly in regions experiencing rapid demographic transition. The marked regional disparities, with particularly high burdens in Oceania, Eastern Europe, and parts of Asia, likely reflect differences in healthcare access, occupational safety regulations, and fall prevention strategies.

The significant regional disparities identified in our study warrant attention. These regional disparities are evidence for the interplay of multiple complex factors that include but are not limited to demographic changes, levels of economic growth, availability of healthcare services, and the transitions in style of life. The increase of forearm fractures in Oceania is particularly apparent, reflecting a 152.48% rise in incidence cases, 164.82% increase in prevalence cases, and 167.17% rise in YLDs numbers, all of which correspond to approximately a 20% increase in rates. Several potential contributing factors may be considered. Lifestyle transitions may have contributed to this increase. For instance, studies suggest that Pacific Island nations have experienced changes in dietary habits from traditional to Western patterns, which may be characterized by reduced intake of calcium-rich traditional foods and could potentially contribute to increased risk of osteoporosis and fractures (29, 30). However, the direct causal relationship between dietary transitions and the observed fracture trends in Oceania requires further investigation (31, 32). Additionally, the availability of better healthcare services and surveillance systems in this region certainly may have contributed to a higher fracture reporting rate (33). Conversely, Central Europe demonstrates epidemiological improvement with both case declines (−32.17% from 1,453,791 to 986,092) and rate declines (−22.81% to 909.72 per 100,000), reflecting successful prevention programs.

The prevalence distribution in females showed a bimodal pattern, peaking in adolescence and post-menopause. This pattern might result from both mechanistic and physiological risk factors. The adolescent peak likely reflects increased physical activity, while the second peak results from fragility fractures associated with osteoporosis, particularly in postmenopausal women. These age-related peaks suggest targeted interventions, such as school-based safety programs for adolescents and osteoporosis screening for postmenopausal women. The observed trends by age and sex are consistent with known fracture epidemiology, where underlying biological mechanisms drive distinct fracture patterns. In females, the dramatic increase in forearm fracture rates after age 50 likely reflects multiple interacting factors, including but not limited to estrogen decline during menopause. Within the first decade post-menopause, this decline accelerates bone resorption and microarchitectural deterioration, reducing bone strength by 20–30% (34). These hormonal changes, coupled with age-related deterioration in neuromuscular coordination and balance, substantially increase fall risk. The distal radius is particularly vulnerable to these physiological changes. And this also explains why it’s often the first fracture site in postmenopausal women. Our findings are consistent with studies reporting that forearm fractures in women over 50 were strongly associated with low bone mineral density (35). The prevalence rates in females were double those in males in older age groups, highlighting a significant gap in osteoporosis screening and management. This gap might be exacerbated in regions where bone density screen scans are limited (36). In contrast, the male predominance in young adulthood appears to reflect different mechanisms, potentially including higher-energy trauma from occupational exposure, risk-taking behaviors, and contact sports that generate greater force transmission to the forearm. However, these patterns may also be influenced by cultural expectations, access to safety equipment, and behaviors that differ between sexes. The relative protection against age-related fractures in older adult men may involve their higher peak bone mass, larger bone size, and slower rate of bone loss compared to women, though lifestyle factors and cultural attitudes may also contribute to these differences (37). However, the contribution of these factors remains unclear and warrants further investigation.

Over the past three decades, Oceania, Western Sub-Saharan Africa, and Central Sub-Saharan Africa have experienced the largest increases in prevalence. These increases were likely due to multiple factors, including improved reporting, demographic transitions, and changes in healthcare access. Conversely, regions such as Central Europe and Eastern Europe showed a decline in prevalence rates, which could reflect successful injury prevention and bone health initiatives. In particular, strategies targeting osteoporosis prevention and post-fracture rehabilitation have been successful in reducing the long-term impact of fractures in some regions (38, 39).

YLDs, which measure the years lived with disability due to the injury, highlight the chronic nature of this injury, including functional limitations and pain. Individuals who suffer from forearm fractures often experience persistent functional limitations even after the bone has healed. The substantial increase in YLD numbers (42.22%) from 1990 to 2021 underscores the growing global impact of forearm fractures. We observed a sevenfold difference in YLDs between females and males in the ≥95 age group (1,139 versus 162). The sex disparities suggest that current prevention strategies may need refinement to address sex-specific risk factors, especially for older women. Similarly, Johansson found that elderly women were at a higher risk of falls compared to men, which may contribute to the higher incidence of fractures and subsequent complications such as chronic pain and reduced mobility (40). Our study found that Oceania, Central Sub-Saharan Africa, and South Asia had the highest increases in YLDs, suggesting a growing burden of disability in these regions. These findings match global trends in musculoskeletal disorders and the growing disability burden from nonfatal injuries (41). These injuries disproportionately affect regions with limited access to quality healthcare. The global decline in YLDs for forearm fractures (−15.69%) reflects advancements in acute care and rehabilitation. The interpretation of our findings requires careful consideration of the relationship between age-standardized rates and absolute counts. While global incidence cases increased by 22.25%, age-standardized rates decreased by 16.75%, indicating population aging as the primary driver. This pattern suggests that prevention strategies might be effective at the individual level; however, healthcare systems must prepare for growing absolute demand. Similarly, despite age-standardized prevalence rates declining by 15.66% and YLDs declining by 15.69%, absolute prevalence cases increased by 39.12%, and the YLDs burden rose by 42.22%. This divergence highlights the need for enhanced healthcare capacity and long-term care planning despite successful prevention efforts. Regional patterns provide additional insights into these trends. Areas like Central Europe and Eastern Europe, which experienced declines in both absolute numbers and age-standardized rates, suggest effective fracture prevention programs that could serve as models for other regions. Conversely, regions such as Oceania, showing increases in both metrics, urgently require targeted interventions. Most concerning are regions with declining age-standardized rates but rapidly increasing absolute cases, as these areas will require expanded healthcare services to meet growing demands.

The causes of forearm fractures identified in our study align with the existing literature. However, it exhibits marked geographic heterogeneity. The predominance of falls as the primary cause across most regions corresponds with previous studies of other types of trauma. In high-income regions, falls predominantly involve domestic incidents among elderly populations, such as slips on stairs or bathroom accidents, often related to age-associated balance and mobility impairments. Several interventions have demonstrated effectiveness in reducing fall-related fractures. Tai Chi programs reduced fall risk by 43% in community-dwelling older adults (42), while home-based exercise programs combining balance and strength training decreased fall-related fractures by 35% (43). In contrast, falls in low-income regions frequently occur in occupational settings, exacerbated by inadequate safety equipment and insufficient workplace regulations. This differentiation underscores the necessity of region-specific preventive strategies. Exposure to mechanical forces, often linked to industrial or workplace environments, was another major contributor. The high rate of mechanical forces in Central and Eastern Europe underscores persistent workplace risks and warrants further emphasis on the need for improved occupational safety measures. Road injuries also significantly impacted regions such as Southeastern Europe, North Africa, the Middle East, and Southern Sub-Saharan Africa. This likely correlates with rapid motorization, insufficient pedestrian infrastructure, and high motorcycle use, as observed in Nigerian trauma studies (44). Interpersonal violence contributed minimally on a global scale, though it was more prominent in Oceania and Southern Sub-Saharan Africa. Animal contact-related injuries were more prevalent in the Caribbean, which may be influenced by local environmental factors. Other causes, such as self-harm and poisonings, played a less prominent role but should not be overlooked in specific regions.

Furthermore, the growing burden in regions with limited healthcare resources emphasizes the importance of developing cost-effective screening tools and preventive interventions suitable for diverse healthcare settings. Additionally, the substantial YLDs burden highlights the importance of addressing post-fracture care quality. Finally, the regional variations in causes suggest that prevention strategies should be tailored to local injury patterns-prioritizing road safety in regions where traffic injuries contribute significantly, workplace safety where mechanical exposures predominate, and falls prevention where this is the primary mechanism.

Our findings have revealed several important clinical and policy implications. First, the substantial increase in forearm fracture burden, signals the need for healthcare systems to prepare for rising demand for orthopedic services, rehabilitation resources, and long-term care. Second, the marked sex disparities in older adults highlight the critical importance of sex-specific preventive strategies, particularly intensified osteoporosis screening and treatment for postmenopausal women. From a policy perspective, our findings underscore the need for region-specific interventions tailored to local epidemiological patterns and healthcare contexts. For Central and Eastern Europe (highest burden regions with >900 per 100,000): Priority should focus on comprehensive fall prevention programs including mandatory home safety assessments for elderly populations, expansion of osteoporosis screening programs, and workplace safety regulations to address the high mechanical force injury rates identified in these regions. For Oceania (152% increase, highest growth globally): Urgent intervention is needed including sports injury prevention programs targeting the identified outdoor activity risks, enhanced healthcare infrastructure development to manage growing caseloads, and dietary intervention programs addressing calcium deficiency in Pacific Island populations. For Sub-Saharan Africa (lowest rates but data limitations): Investment in healthcare infrastructure and surveillance systems is needed, along with basic fracture prevention education adapted to local cultural contexts and resource constraints. For osteoporosis management in high-burden regions, we recommend: (1): expand access to bone mineral density testing through dual-energy X-ray scans, particularly for women aged 65+, men over 70, and individuals with prior fragility fractures; (2) ensure reimbursement for approved osteoporosis treatments including bisphosphonates, denosumab, and anabolic agents; (3) enhance healthcare professional education through targeted training programs; (4) promote public awareness campaigns about osteoporosis risk factors and prevention strategies; and (5) integrate multidisciplinary care approaches involving endocrinologists, primary care physicians, and nutritionists to manage osteoporosis, emphasizing nutritional interventions such as calcium and vitamin D supplementation. Given the disproportionate burden among postmenopausal women, sex-specific approaches are essential, particularly in regions with the highest prevalence rates such as Eastern Europe. Additionally, in areas where mechanical forces are prominent injury causes, enhancing workplace safety regulations through mandatory protective equipment use, regular training, and safety audits can significantly reduce fracture incidence.

The 95% UIs presented in this study highlight the variability and precision of our estimates. The overlap of uncertainty intervals across different countries or regions indicates that observed differences might not always represent statistically significant distinctions. Consequently, caution should be exercised in interpreting regional comparisons when intervals substantially overlap. Additionally, the Bayesian meta-regression model underlying these estimates depends on certain assumptions, and variations in these assumptions could influence results.

### Limitations

Several limitations of this study should be noted. First, the GBD modeling approach generates estimates for many countries through statistical modeling rather than direct observation, particularly in low-income regions with limited surveillance systems. The quality and completeness of injury reporting vary across regions, particularly in areas with limited surveillance systems. Underreporting in low-income countries where fractures often go undiagnosed likely leads to underestimation. For example, lower completeness in injury coding in Sub-Saharan Africa compared to regions like Western Europe or high-income North America could lead to underestimation of regional ASRs. Furthermore, diagnostic biases resulting from limited access to radiographic equipment or skilled medical personnel in low-income countries may skew prevalence data, potentially leading to systematic underestimation of the true disease burden. Additionally, the modeling framework assumes consistent relationships between covariates (such as socioeconomic factors and healthcare access) and fracture rates across diverse healthcare systems and cultural contexts, which may not fully capture unique local risk factors. Caution should be exercised when interpreting these regional estimates. However, these limitations do not fundamentally undermine the study’s primary conclusions. Second, the study did not account for treatment data across regions, which might influence disability outcomes. Forearm fracture management varies widely across healthcare systems, and our YLDs estimates do not include these differences. Third, the ICD-10 S52 classification lacks specificity regarding distinct fracture patterns and exact anatomical locations within the radius and ulna, potentially leading to coding inaccuracies and misclassification bias. Lastly, our analysis is limited by the lack of sensitivity testing. Future studies incorporating sensitivity analyses would be beneficial to assess the robustness of findings and enhance confidence in interpretations.

## Conclusion

This study provides the first comprehensive analysis of global forearm fracture incidence, prevalence, and disability burden using GBD data from 1990 to 2021. The incidence, prevalence, and YLDs of forearm fractures have increased in absolute numbers but declined in age-standardized rates over the past three decades. We identified notable regional and sex disparities. Falls remained the primary cause of these fractures, particularly in Central and Eastern Europe, while exposure to mechanical forces and road injuries also contributed significantly in certain regions. Policymakers should implement interventions including osteoporosis screening and mobile applications for fall risk assessment. High-incidence regions require comprehensive fall prevention programs and mandatory screening, while emerging high-burden areas need infrastructure development. Health systems must establish fracture services, mandate prevention program coverage, and implement real-time monitoring with adaptive management to ensure measurable population impact.

## Data Availability

The datasets generated and/or analyzed during the current study are available from the corresponding author on reasonable request.

## Footnotes

^1^ https://vizhub.healthdata.org/gbd-results
