# Supplementary material for: The incidence, prevalence, and years lived with disability of forearm fractures: a systematic analysis based on the global burden of disease study 2021
Source: Front Public Health. 2025 Jul 10;13:1598660. doi: 10.3389/fpubh.2025.1598660 (PMC12287112; doi:10.3389/fpubh.2025.1598660)
Supplement: Supplementary file 1 [file Table_1.docx]

Supplementary Table 1. Incidence cases of forearm fractures in 1990 and 2021 and the percentage change in the age-standardized rates (ASRs) per 100,000 population, for both sexes in 204 countries and territories.

| Country | 1990 | | 2021 | | Percentage change in  the ASRs per 100,000 |
| --- | --- | --- | --- | --- | --- |
|  | No  (95% UI) | ASRs per 100,000  (95% UI) | No  (95% UI) | ASRs per 100,000  (95% UI) |  |
| Global | 26098810  (20967988,32372267) | 483.3  (387.4,599.4) | 31905396  (25403829,39982115) | 402.3  (319.9,505.2) | -16.7  (-19.4,-14.2) |
| Taiwan  (Province of China) | 87431  (71131,108787) | 417.5  (339.4,520.8) | 55783  (44324,70221) | 218.5  (175.5,271.9) | 9.4  (3.9,15.2) |
| People's Republic of China | 4386645  (3532947,5478662) | 369.6  (296.7,461) | 5790636  (4502029,7414711) | 404.5  (317.1,516.5) | -22.2  (-26.1,-17.5) |
| Kingdom of Cambodia | 38155  (31340,46303) | 361.6  (298.2,434.4) | 64856  (51576,82506) | 386.6  (305.8,491.6) | -25  (-28,-22.3) |
| Lao People's Democratic Republic | 14279  (11756,17951) | 332  (275.8,408.9) | 19361  (15998,23270) | 259.1  (215.2,312.9) | 1  (-8.7,11.9) |
| Democratic People's Republic of Korea | 53883  (44913,65134) | 257.4  (214.3,312.8) | 55179  (46299,65895) | 200.2  (168.7,237.7) | 6.9  (-3.6,16.2) |
| Republic of Indonesia | 692587  (564743,832366) | 368.4  (301,444) | 750997  (615269,910997) | 276.1  (226,336.4) | **-36.1**  **(-57.2,-15.4)** |
| Malaysia | 48717  (40194,58164) | 277.3  (230,330.6) | 89549  (73575,108487) | 276.7  (226.3,336.5) | -11.7  (-17,-6.6) |
| Republic of Maldives | 755  (601,910) | 336.4  (273,405.8) | 1594  (1273,1992) | 304.2  (242,382.2) | -5.8  (-10.4,-1) |
| Republic of the Union of Myanmar | 167733  (138978,200751) | 397.2  (330.2,475.1) | 227623  (184111,280946) | 401.2  (324.9,496.3) | -5.6  (-17.5,3) |
| Democratic Socialist Republic of Sri Lanka | 99869  (75682,144887) | 546.6  (418.8,768.3) | 91461  (72910,114230) | 405.7  (324.1,506.1) | -9.6  (-17.6,-1.5) |
| Kingdom of Thailand | 231830  (194327,271558) | 388.4  (327,456.4) | 239414  (195507,293494) | 359.9  (296.4,440) | 4.6  (0.4,9.4) |
| Republic of the Philippines | 239888  (198839,293439) | 364.9  (304.7,440.3) | 297447  (242841,362957) | 259.1  (212.1,316.2) | -7.3  (-14.7,-0.7) |
| Democratic Republic of Timor-Leste | 3334  (2476,5175) | 395.9  (301,594.8) | 3599  (2986,4379) | 253  (210,305.4) | -28.7  (-31.1,-26) |
| Republic of Fiji | 1917  (1555,2393) | 240.2  (196,295.2) | 2082  (1693,2607) | 226.2  (184.8,282.1) | -0.2  (-5.1,4.5) |
| Socialist Republic of Viet Nam | 216098  (177239,258571) | 327.1  (269.8,392.8) | 366227  (295327,446385) | 371.3  (299.7,456.9) | 24  (16.9,30.7) |
| Independent State of Papua New Guinea | 12320  (9916,15400) | 303.9  (247.1,378) | 38238  (30689,48474) | 376.7  (302.3,475.5) | -9.8  (-17,-2) |
| Republic of Kiribati | 154  (128,186) | 199.6  (165.9,239.7) | 220  (179,271) | 176.3  (144.9,216.1) | -17.2  (-20.2,-13.7) |
| Republic of the Marshall Islands | 123  (101,151) | 273.7  (229.9,330.5) | 155  (128,192) | 271.8  (224.4,337) | -1.7  (-5.9,2.6) |
| Federated States of Micronesia | 289  (240,347) | 281.9  (236.7,336.6) | 307  (255,379) | 296.7  (245.8,365.7) | **-45.8**  **(-48.5,-42.8)** |
| Independent State of Samoa | 518  (425,644) | 295.6  (242.6,360.2) | 585  (471,733) | 279  (227.1,348.5) | -25.8  (-48.4,-4.7) |
| Kingdom of Tonga | 252  (206,313) | 252.9  (209.6,310.1) | 234  (187,294) | 221.1  (178,277.9) | 15  (8.6,21.8) |
| Solomon Islands | 1276  (1030,1603) | 417.4  (338.5,518.1) | 3238  (2539,4138) | 506  (394.5,640.2) | **-47.7**  **(-50,-45.1)** |
| Republic of Vanuatu | 376  (305,463) | 245.2  (201.3,299.8) | 756  (619,938) | 241.1  (198.4,297.9) | 5.3  (0,10.3) |
| Republic of Azerbaijan | 41130  (33933,49019) | 520.7  (429.8,619.7) | 44157  (35736,53568) | 431.4  (348.5,524.8) | -12.6  (-18.3,-7.7) |
| Republic of Armenia | 27649  (22712,33612) | 782.6  (641,955) | 11793  (9709,14094) | 424.3  (345.5,510.6) | -21.9  (-33.6,-12.9) |
| Georgia | 43617  (34914,53477) | 808.6  (645.6,995.7) | 27408  (22002,33742) | 845.9  (677,1042.3) | -29  (-35,-24.6) |
| Republic of Kazakhstan | 131031  (107304,158277) | 765.7  (627.5,923.4) | 125678  (100773,154076) | 667.6  (534.6,819.2) | -28.6  (-31.5,-25.4) |
| Republic of Tajikistan | 35468  (28775,43529) | 595.9  (486.9,723.8) | 45870  (36926,55980) | 425.1  (343.5,517.1) | **-35.2**  **(-38,-31.8)** |
| Kyrgyz Republic | 33396  (27070,41135) | 695.4  (566.1,853.7) | 32164  (26189,38802) | 451  (367.8,541.1) | -19.6  (-22.4,-16.7) |
| Mongolia | 14396  (11895,17250) | 617.3  (513.1,733.1) | 24211  (19663,29722) | 710.2  (575.3,872.3) | 13.5  (5.4,21.9) |
| Turkmenistan | 22807  (18459,27825) | 540.7  (441,651) | 22961  (18489,27724) | 431.8  (348,521.6) | -19.6  (-26.8,-12.4) |
| Republic of Albania | 35976  (28416,45094) | 1019.1  (802.4,1278) | 22058  (17688,27292) | 919.2  (724.3,1145.6) | -0.7  (-5.2,4) |
| Republic of Uzbekistan | 136181  (109861,166003) | 582  (475.5,703.7) | 162567  (132818,196061) | 467.9  (381.9,565.8) | 21.2  (14.8,28.1) |
| Bosnia and Herzegovina | 45568  (35972,57149) | 1001.8  (789.2,1252.4) | 23313  (18503,28675) | 817.6  (639.8,1012.7) | -19.5  (-24.1,-15.2) |
| Republic of Bulgaria | 99706  (80013,120508) | 1246.8  (995.5,1512) | 57315  (45312,70166) | 1004.3  (788.3,1234.3) | -21.1  (-24.8,-17.3) |
| Republic of Croatia | 56354  (45467,68304) | 1188.4  (956.7,1445.5) | 42849  (33154,54699) | 956  (734,1203.8) | -22.3  (-25.7,-19.4) |
| Hungary | 134633  (106297,166741) | 1247.7  (992.6,1534.3) | 84938  (66335,105267) | 904.5  (703.8,1129.6) | -12.8  (-16.3,-9.3) |
| Czech Republic | 141494  (113201,174194) | 1355.9  (1085.4,1664.8) | 97405  (77053,120287) | 968.7  (763.2,1195.1) | **-40.5**  **(-42.9,-37.9)** |
| North Macedonia | 18688  (15171,22909) | 931.6  (755.1,1138.2) | 16075  (12752,19740) | 814.6  (643.5,1002.6) | -20.1  (-24.2,-15.3) |
| Montenegro | 6676  (5309,8228) | 1062.2  (844.8,1310.1) | 5198  (4102,6392) | 905.6  (709.8,1130.1) | -20.7  (-24.4,-17) |
| Romania | 291568  (233435,358953) | 1286  (1029.6,1586.3) | 160260  (126869,196512) | 944.8  (746.7,1163.5) | -14.7  (-17.1,-12.3) |
| Republic of Serbia | 85458  (68604,103298) | 926.4  (740.4,1122.3) | 66176  (52532,81564) | 809  (633.6,1001.9) | **-37.7**  **(-40.8,-34.8)** |
| Republic of Poland | 418012  (337865,507993) | 1100.1  (882.7,1340.6) | 316484  (249898,389255) | 854.4  (676.8,1056.1) | -2.1  (-6.8,2.5) |
| Slovak Republic | 66828  (52936,82261) | 1257.2  (994.9,1549.6) | 55319  (43412,68523) | 1059  (825.7,1314.3) | -18.4  (-25.2,-10.8) |
| Republic of Slovenia | 29583  (23358,36478) | 1486.6  (1171.1,1831.2) | 24347  (19030,30606) | 1172.8  (916.7,1469.7) | -15.8  (-18.6,-13) |
| Republic of Belarus | 105234  (85450,129030) | 1037.1  (839.3,1274.9) | 89058  (70288,111900) | 1015.6  (803.3,1282.9) | -19.2  (-23.7,-15.1) |
| Republic of Estonia | 19347  (15561,23912) | 1264.4  (1016.7,1562.3) | 9372  (7451,11744) | 787.5  (620.6,994.3) | -12.6  (-21.6,-5.1) |
| Republic of Moldova | 46857  (38213,56860) | 1052.6  (857.2,1279.6) | 21887  (17735,26933) | 661.8  (533.2,818.2) | -12.7  (-17.5,-7.9) |
| Republic of Latvia | 36574  (29380,44870) | 1395.7  (1119.9,1712.3) | 14396  (11464,17715) | 830.1  (661.7,1028.8) | -25.7  (-28.2,-23.4) |
| Republic of Lithuania | 45750  (36744,56311) | 1246.5  (1004,1538.8) | 22987  (18082,29104) | 871.4  (695.2,1102.8) | -4.7  (-9.1,-0.2) |
| Russian Federation | 1726495  (1427277,2070674) | 1167.7  (952.3,1400.8) | 1288347  (1039715,1588734) | 943  (754.9,1171.4) | -27.5  (-31.2,-23.7) |
| Ukraine | 617351  (491035,777915) | 1222.7  (968.9,1543.4) | 384062  (308480,477781) | 969.5  (776.6,1205.1) | **-37.1**  **(-39.8,-34.3)** |
| Brunei Darussalam | 1089  (830,1406) | 397.2  (302.6,512.3) | 1578  (1188,2050) | 345.3  (257.9,447.1) | -13.1  (-17.7,-8.7) |
| Japan | 451832  (335966,593466) | 370.3  (274,486.5) | 328507  (241264,437150) | 274.9  (201.7,363.7) | -20.3  (-24.8,-15.5) |
| Republic of Korea | 266471  (201812,348215) | 575.4  (435.2,747.7) | 213144  (155280,284795) | 406.6  (293.5,541.8) | -9.9  (-13.6,-6) |
| Republic of Singapore | 11529  (8665,15093) | 357.8  (266.2,466.3) | 16245  (11875,21710) | 327.9  (238.9,436.7) | -26.5  (-29.5,-23.7) |
| Australia | 98053  (72484,127426) | 608  (446.2,796.5) | 142843  (104811,189244) | 579.7  (417.4,776.4) | 5.1  (0.6,9.3) |
| New Zealand | 23887  (17910,30966) | 706.7  (524.8,916.1) | 31534  (23181,41551) | 665.5  (478,889.7) | -8.5  (-12.9,-4.3) |
| Republic of Austria | 49543  (35848,65609) | 634.7  (458.4,844) | 44631  (31787,60730) | 505.8  (357.7,695.1) | -13  (-18.1,-8.5) |
| Principality of Andorra | 331  (233,450) | 622.3  (442.4,846.2) | 590  (417,811) | 654.3  (458,901.7) | **-30.1**  **(-32.8,-27.4)** |
| Kingdom of Belgium | 57833  (41477,78350) | 593.1  (428.5,803.3) | 71876  (50709,97505) | 589.2  (413.4,802.9) | -14.9  (-20.4,-10.2) |
| Republic of Cyprus | 3892  (2827,5153) | 507.6  (368.2,673.8) | 6270  (4455,8460) | 486.1  (344.6,662.3) | -24.4  (-28.1,-20.7) |
| French Republic | 357533  (260188,477916) | 608.6  (441.7,809) | 379062  (268660,514773) | 543.6  (381,742.8) | **-35.3**  **(-39.6,-30.9)** |
| Kingdom of Denmark | 26750  (19314,35789) | 499.8  (354.1,668.5) | 23751  (16625,32305) | 425.2  (296.1,586.6) | -18.3  (-23,-13.1) |
| Republic of Finland | 34546  (24651,46378) | 707.4  (501.5,966.3) | 34916  (24606,47305) | 637.2  (444.6,886.3) | -8.4  (-12.5,-3.7) |
| Federal Republic of Germany | 386141  (278638,516560) | 493.8  (356.5,666.1) | 387243  (274984,526756) | 452.5  (318.3,618.9) | -5.8  (-9.3,-2.9) |
| Ireland | 16489  (11933,22087) | 457.8  (332.4,612.9) | 21049  (14865,28876) | 456.4  (319.6,628.5) | -4.2  (-9.6,0.7) |
| Hellenic Republic | 53539  (38845,71480) | 538.3  (389.9,722.3) | 35034  (25294,46408) | 407.1  (290.9,553.3) | -6  (-13.4,0.6) |
| Republic of Iceland | 1266  (909,1703) | 499  (356.6,674.4) | 1510  (1067,2055) | 456.6  (317.8,624.6) | -8.4  (-13,-4.3) |
| State of Israel | 20098  (14553,26710) | 394.4  (287.2,523.7) | 36081  (25836,48992) | 382.9  (274.7,519.8) | -2.9  (-7.7,1.7) |
| Republic of Italy | 350953  (246154,474149) | 616.5  (441.2,833) | 274850  (192521,376198) | 503.6  (356.3,698.4) | -16.3  (-20,-12.7) |
| Kingdom of the Netherlands | 51837  (37555,69255) | 353.1  (254.4,472.9) | 74678  (53144,102032) | 383.2  (270.1,519.8) | -3.5  (-6.8,0.4) |
| Grand Duchy of Luxembourg | 2231  (1642,2978) | 596.4  (438.2,793.9) | 3228  (2275,4396) | 519.1  (366.4,710.2) | -23.3  (-27.2,-19.6) |
| Republic of Malta | 1823  (1299,2475) | 510.8  (363.6,700) | 1937  (1377,2643) | 491.9  (346.2,676.4) | -29.3  (-34.9,-24.6) |
| Kingdom of Norway | 22474  (16299,30061) | 505.5  (365.8,673.3) | 24477  (17472,33643) | 423.2  (296.9,581.3) | 21.5  (13.7,30.5) |
| Portuguese Republic | 48533  (36466,63371) | 493.1  (371.4,644.6) | 32806  (23970,43653) | 318.9  (231.1,426.5) | -5.8  (-11.9,1.7) |
| Kingdom of Sweden | 40760  (29014,54767) | 477  (339.7,641.7) | 43325  (30549,59617) | 400.9  (282.2,552.6) | -0.7  (-5.3,3.8) |
| Kingdom of Spain | 159194  (118348,209103) | 417.8  (312.3,549.6) | 178959  (127682,242957) | 443.2  (314.4,609.9) | 8.5  (3,13.8) |
| Swiss Confederation | 52389  (38076,71002) | 753.8  (540,1014) | 51601  (36443,70783) | 578.2  (408.2,794.5) | -0.3  (-5.9,5.2) |
| United Kingdom of Great Britain and Northern Ireland | 225253  (160588,302292) | 407.6  (293.2,550.9) | 259527  (181058,355369) | 383.8  (270.5,526.1) | -7.8  (-13.5,-2.8) |
| Republic of Chile | 51044  (38329,65832) | 374.9  (281.3,483.3) | 80916  (59167,106208) | 455.6  (331.2,599) | -16  (-19.3,-13.1) |
| Argentine Republic | 113613  (85912,144259) | 341.2  (258.4,434.1) | 146038  (110347,185795) | 329.4  (247.3,421.3) | -10.7  (-15.3,-6.7) |
| Eastern Republic of Uruguay | 12972  (9709,17183) | 419.1  (314.7,556.1) | 13043  (9740,16829) | 393.8  (291.5,509) | -3.7  (-7.1,-0.3) |
| Canada | 89631  (67635,116442) | 317.4  (239.9,411.9) | 133923  (98662,182034) | 292.6  (217.6,387.9) | 5.8  (1.3,10.3) |
| Antigua and Barbuda | 229  (184,286) | 367.7  (296.3,457.9) | 339  (264,428) | 395.5  (311.8,504.9) | 19.1  (15,23.7) |
| Barbados | 719  (596,873) | 283  (234.2,345.9) | 821  (675,1003) | 299.5  (244,368.6) | -16  (-21.6,-10.3) |
| United States of America | 941217  (706485,1229537) | 366.9  (275.5,478.1) | 1202247  (899155,1618912) | 308.1  (229.8,404.4) | 6.1  (-4,15.2) |
| Belize | 692  (566,837) | 332.2  (275.7,398.8) | 1693  (1400,2054) | 376.9  (311.5,456.6) | -0.8  (-5.8,4) |
| Commonwealth of the Bahamas | 824  (688,977) | 305.8  (256.3,360.6) | 1232  (1027,1452) | 322.5  (267.9,380) | 5.5  (1.3,10) |
| Republic of Cuba | 49140  (40351,59058) | 447.4  (364.6,537.6) | 61214  (48306,76065) | 504.6  (406.2,621.2) | -0.7  (-4,2.9) |
| Commonwealth of Dominica | 213  (176,253) | 280.6  (232.9,334.5) | 193  (161,230) | 296.6  (245.5,351.9) | 7.6  (2.4,12.9) |
| Grenada | 306  (252,368) | 339.9  (281.1,407.7) | 376  (302,462) | 371.7  (299.5,454.7) | 5.7  (1.8,9.8) |
| Dominican Republic | 22563  (18775,26857) | 292.3  (243.5,347.8) | 38851  (32237,46429) | 348.3  (288.9,414.8) | -10.7  (-23.4,-0.5) |
| Republic of Guyana | 3018  (2520,3578) | 371.8  (310.4,439.4) | 3299  (2755,3916) | 426.6  (357.2,507.1) | 16  (0.7,46.9) |
| Republic of Haiti | 25762  (21589,30715) | 377.8  (317.1,448) | 59441  (48536,76242) | 438.4  (359.7,557.4) | -21  (-32.8,-12.7) |
| Saint Lucia | 449  (371,540) | 311.3  (259.8,372.5) | 558  (459,676) | 333  (273.2,402.8) | -11.3  (-14.8,-7.4) |
| Jamaica | 8588  (6934,10592) | 343  (276.9,423.7) | 9426  (7642,11566) | 340.4  (275.9,414) | 7.1  (3.3,10.8) |
| Saint Vincent and the Grenadines | 370  (303,446) | 317  (262.8,378.9) | 394  (322,478) | 353.5  (287.4,430) | -24.1  (-26.2,-21.6) |
| Republic of Suriname | 1185  (987,1411) | 290  (243.7,344.9) | 1772  (1466,2104) | 310.7  (256.9,368.5) | -11  (-15.8,-5.9) |
| Republic of Trinidad and Tobago | 4137  (3410,4967) | 329.3  (272.8,391.7) | 4214  (3513,5030) | 327  (270.3,392.2) | -23  (-27.9,-17.8) |
| Plurinational State of Bolivia | 28395  (23355,33880) | 424.3  (350.9,503.2) | 45079  (36701,54747) | 377.9  (307.2,457.4) | 2.9  (-1.4,7.1) |
| Republic of Colombia | 227072  (185355,278808) | 649.8  (531.6,793) | 208909  (167564,257185) | 449.7  (355.9,563.3) | **-30.8**  **(-34.6,-26.8)** |
| Republic of Ecuador | 46757  (38067,56887) | 444.5  (365,535) | 84316  (68145,104205) | 457.3  (371.3,564.7) | 14.7  (10.1,19.8) |
| Republic of Peru | 99321  (82264,121862) | 424.8  (351.8,515.6) | 139327  (112293,169096) | 379.2  (305.4,461) | -8.9  (-11.8,-6.2) |
| Republic of Costa Rica | 16927  (12996,21882) | 524.3  (405.8,669.5) | 21439  (16839,27308) | 477.5  (371.4,609.1) | 12.8  (7,18.3) |
| Republic of El Salvador | 36673  (29299,47105) | 613.1  (494.2,770.6) | 31579  (25695,39035) | 484.7  (393.9,600.1) | 11.5  (6.9,16.4) |
| Republic of Guatemala | 57169  (45257,71330) | 609.4  (492.4,754.6) | 93670  (75151,114947) | 572.3  (460.7,697.8) | **-36.8**  **(-38.3,-35.2)** |
| United Mexican States | 759958  (590925,957995) | 827  (654.8,1030.4) | 671488  (530926,837401) | 522.7  (411.8,652.5) | -27.1  (-31.2,-23.3) |
| Republic of Nicaragua | 20904  (16163,26419) | 451.2  (358.7,553.5) | 26719  (20917,33795) | 400.4  (312.7,504) | 9.4  (2.9,15.3) |
| Republic of Honduras | 27507  (22123,34010) | 499.8  (410.7,603.8) | 44824  (36846,54012) | 429.7  (352.4,516.2) | **-38.7**  **(-49.7,-30.5)** |
| Republic of Panama | 12625  (9946,15877) | 489.7  (388.8,612.6) | 17892  (14126,22402) | 421.1  (331.3,528.9) | 13.5  (7.4,19.3) |
| Federative Republic of Brazil | 1221434  (966028,1546937) | 775  (616.9,978.6) | 1286168  (1022839,1608154) | 587.9  (464.4,741.9) | 7  (1.9,12.1) |
| Bolivarian Republic of Venezuela | 126235  (99037,157971) | 608  (482.8,753.9) | 133234  (108540,163435) | 527.5  (426,651.2) | -14  (-17.5,-10.6) |
| Republic of Paraguay | 24721  (19130,31378) | 562.7  (446.4,711.1) | 37271  (29740,46161) | 503.2  (402,622.8) | 7.7  (-1.8,22.2) |
| People's Democratic Republic of Algeria | 151450  (124650,184915) | 549.7  (455,662.1) | 187245  (151873,231566) | 423.3  (342.7,525.5) | -13.2  (-16.5,-9.5) |
| Kingdom of Bahrain | 2073  (1696,2496) | 374.7  (307.4,448.8) | 5760  (4670,7081) | 368.1  (298.8,456.7) | -8  (-14.6,-2.2) |
| Arab Republic of Egypt | 280985  (234944,334423) | 463  (387.9,550.7) | 396423  (327799,478410) | 366.3  (303.7,440.3) | -20.9  (-25.5,-16) |
| Islamic Republic of Iran | 428969  (347619,538365) | 689.7  (562.9,863.6) | 358344  (295400,435747) | 423.1  (347,516.4) | -6.1  (-15.9,1.3) |
| Hashemite Kingdom of Jordan | 17580  (14373,21483) | 421.5  (348.5,509.4) | 45818  (36585,57103) | 349.4  (280.5,432.8) | -6.3  (-13.9,1.8) |
| Republic of Iraq | 137356  (112509,163921) | 693.7  (575.4,827.1) | 220767  (179991,263899) | 505.9  (413.7,604) | -10.6  (-14.2,-6.9) |
| State of Kuwait | 12949  (9902,18065) | 681.8  (520.7,978.9) | 21761  (17258,27318) | 453.4  (359.8,575.2) | -37  (-50.1,-23.6) |
| Kingdom of Morocco | 148635  (121746,179013) | 550.2  (453.5,659.2) | 175255  (141315,214284) | 468.9  (379.4,572.9) | -11.9  (-16.1,-8.4) |
| Lebanese Republic | 17371  (13941,22363) | 588.9  (465.5,745.7) | 20697  (16704,25680) | 371.2  (296.6,460.3) | -14  (-17.1,-10.9) |
| Palestine | 11221  (8949,14238) | 490.1  (394.3,615.9) | 23507  (18860,29435) | 422.8  (341.2,526.6) | -10.6  (-15.2,-5.5) |
| State of Libya | 21199  (17205,25889) | 468.4  (385.1,565.5) | 33403  (28014,40579) | 504.7  (419.1,616.2) | -1.8  (-7.6,3.9) |
| Sultanate of Oman | 13525  (11056,16280) | 666.7  (548,801) | 25287  (20698,31122) | 541.4  (442,670.9) | -18.8  (-24.9,-12.1) |
| State of Qatar | 2894  (2378,3488) | 569.2  (465.2,684.1) | 17326  (13910,21593) | 523.6  (419.2,657.9) | **-33.5**  **(-53,-18.1)** |
| Syrian Arab Republic | 57748  (45880,73106) | 405.1  (325.8,502.6) | 64240  (50712,82954) | 452.2  (359.3,577.9) | -13.4  (-16.7,-10.2) |
| Republic of Tunisia | 44184  (35801,53491) | 493.6  (401.6,594.9) | 51061  (41339,62048) | 441.5  (356.7,537.8) | -3  (-7.7,2) |
| Kingdom of Saudi Arabia | 158477  (126687,197792) | 971.6  (781.8,1200.7) | 464989  (364227,586395) | 1068.9  (842.6,1347) | -17.1  (-22.5,-11.9) |
| Republic of Turkey | 255982  (208817,308991) | 415.3  (340.4,496.5) | 318576  (252631,396984) | 389.2  (308,486) | 11.6  (2.4,23.1) |
| Republic of Yemen | 64797  (53783,78072) | 440.5  (370.3,525.7) | 196477  (152374,285054) | 537.5  (423.1,762.2) | 29.4  (20.1,39.5) |
| United Arab Emirates | 11816  (9680,14189) | 581.1  (475.3,703.5) | 55269  (44702,68141) | 547.6  (441,679.7) | -13.7  (-23.9,-6) |
| Kingdom of Bhutan | 1884  (1492,2309) | 291  (235.6,351.1) | 2797  (2237,3501) | 376.7  (301.5,472.5) | **37.3**  **(5.4,96.5)** |
| Islamic Republic of Afghanistan | 50208  (41703,60342) | 477.5  (402.2,571.6) | 230302  (162790,383454) | 655.5  (473.6,1058.2) | 22  (-0.8,74.4) |
| Republic of India | 4270764  (3414498,5428599) | 520.3  (415.5,658.2) | 6398991  (5082187,8144972) | 458.3  (361.4,586.8) | **-53.8**  **(-67.5,-41.4)** |
| People's Republic of Bangladesh | 269501  (218549,335060) | 223.7  (182.8,276.8) | 402372  (317710,512391) | 236  (187.2,299.7) | -14.8  (-20,-10.1) |
| Federal Democratic Republic of Nepal | 93371  (72274,122451) | 474.3  (369.1,614.2) | 166948  (127146,219040) | 521.5  (399,683.2) | 5.5  (-1.2,12.7) |
| Islamic Republic of Pakistan | 283456  (229282,354565) | 245.3  (201.9,300.6) | 515491  (427343,629560) | 212.9  (178.7,256.5) | -12.5  (-16.1,-8.9) |
| Republic of Angola | 35252  (27984,47857) | 315.7  (254.9,417) | 71789  (59423,88919) | 220.4  (182.8,267) | -0.5  (-5.3,4.5) |
| Central African Republic | 6754  (5700,7981) | 241.4  (203.4,284.4) | 16557  (13421,21767) | 286.2  (236.1,362.9) | **-30.2**  **(-46.9,-17.5)** |
| Democratic Republic of the Congo | 93632  (78314,111597) | 231.9  (194,275.7) | 202383  (167516,245283) | 224.8  (187.3,269) | -13.2  (-16.1,-10.3) |
| Republic of Equatorial Guinea | 1029  (871,1225) | 235.6  (198.3,278.3) | 2954  (2399,3634) | 200.2  (165,242.9) | 10  (3.5,16.1) |
| Republic of the Congo | 5596  (4670,6697) | 234.1  (196.9,277.4) | 10792  (8987,13165) | 204.8  (171.3,249) | **-67.3**  **(-83.4,-43.6)** |
| Gabonese Republic | 2493  (2080,2996) | 253  (211.2,301.7) | 4034  (3334,4925) | 227.4  (188.2,278.5) | -5.8  (-10.8,-0.8) |
| Union of the Comoros | 1080  (903,1305) | 228.1  (192.2,268.8) | 1536  (1279,1848) | 209.2  (175.9,249.2) | -10.1  (-13.6,-7) |
| Republic of Burundi | 13874  (11510,16634) | 242.5  (201.3,288.2) | 28147  (23083,34379) | 210  (173.1,252.8) | -20  (-34.1,-8.5) |
| Republic of Djibouti | 1097  (885,1386) | 250.3  (207.1,308) | 2605  (2166,3116) | 214.1  (178.9,256.1) | 18.6  (4.2,48.8) |
| State of Eritrea | 27676  (15504,57130) | 701.2  (408.4,1370.6) | 14481  (12102,17244) | 228.9  (192.9,272) | -7.7  (-11.5,-4) |
| Federal Democratic Republic of Ethiopia | 280901  (196042,458910) | 514  (373.2,799.1) | 257627  (206586,337991) | 237.3  (193.9,299) | -14.5  (-27.7,-5.6) |
| Republic of Kenya | 51804  (42676,62591) | 229.4  (191.3,272.6) | 100172  (82860,120298) | 215.6  (179.2,259.3) | 9.9  (4,15.5) |
| Republic of Madagascar | 27913  (23110,33806) | 218.1  (180.2,259.6) | 53235  (43464,64909) | 182.7  (150.8,219.9) | **-33.3**  **(-35.6,-30.9)** |
| Republic of Malawi | 22455  (18481,27177) | 212.3  (174.6,253.2) | 38626  (30979,47863) | 195.9  (159.9,238.6) | -11  (-15.8,-6.8) |
| Republic of Mauritius | 2870  (2355,3478) | 248.9  (204.9,299.3) | 3091  (2491,3814) | 247.8  (200,309) | -2.9  (-6.8,0.8) |
| Republic of Rwanda | 26177  (20012,38462) | 336  (262,467.7) | 27012  (22015,32740) | 205.2  (169.6,247.2) | -16.2  (-19.4,-13) |
| Republic of Seychelles | 218  (180,262) | 293  (242.7,350.4) | 274  (224,337) | 260.7  (213.1,323) | 0  (-5,4.8) |
| Republic of Mozambique | 38001  (30279,50526) | 262.8  (210.6,337.3) | 72460  (58764,88578) | 232.1  (192.9,278.6) | -15  (-21.3,-9.3) |
| Federal Republic of Somalia | 27026  (20477,40660) | 321.8  (250,459.8) | 55790  (45855,68376) | 257.4  (216.2,308.7) | -6  (-8.8,-3.3) |
| Republic of Uganda | 41085  (33689,49934) | 221.6  (184.5,266.8) | 91385  (74435,112761) | 207.8  (172.4,251.8) | 0.5  (-3.5,4.2) |
| Republic of Zambia | 18954  (15686,22930) | 226.1  (189.1,269.3) | 42399  (34592,52537) | 226  (187,277.1) | -3.5  (-11.1,1.5) |
| United Republic of Tanzania | 63265  (52161,76287) | 228.1  (190.7,272.9) | 129372  (105569,158979) | 216.6  (178.7,264.4) | -8.3  (-12,-4.7) |
| Republic of Botswana | 3320  (2741,3981) | 246.3  (205.9,292.2) | 6475  (5450,7734) | 263.7  (221.3,315.3) | -1.7  (-5,1.6) |
| Republic of Namibia | 3366  (2819,4001) | 237.5  (201,280.5) | 5932  (4970,7093) | 238.7  (200.8,282.7) | -6.3  (-13.7,-0.8) |
| Republic of South Africa | 146520  (122664,174993) | 382  (320.3,457) | 150459  (126586,179272) | 254.9  (213.5,304.3) | **-38.9**  **(-57.1,-23.3)** |
| Kingdom of Lesotho | 3684  (3102,4387) | 235.6  (200.5,277.4) | 5506  (4664,6556) | 285.4  (243.1,337.4) | 7.1  (2.8,11.9) |
| Kingdom of Eswatini | 2107  (1773,2511) | 258.3  (218.5,304.6) | 3329  (2815,3964) | 275.5  (232.7,328.4) | -11.9  (-16.8,-6.7) |
| Republic of Benin | 12253  (10175,14706) | 236.6  (198.4,281) | 31353  (25854,38144) | 229.7  (191.6,275.2) | -0.9  (-5.9,3.7) |
| Republic of Zimbabwe | 23280  (19261,27973) | 227.6  (190.3,269.6) | 32670  (27367,39306) | 213.2  (180.6,251.9) | -11.7  (-25,-3.1) |
| Burkina Faso | 24010  (19888,28789) | 242.7  (204.1,287.6) | 59661  (48889,72107) | 257.2  (213.8,308.7) | 21.1  (15.3,27.9) |
| Republic of Cameroon | 23142  (19066,28275) | 219.6  (181.1,265.7) | 73213  (59730,90464) | 231  (190.8,281.3) | -6.3  (-9,-3.6) |
| Republic of Chad | 15976  (13069,20022) | 245.1  (202.9,300.9) | 43732  (36205,52903) | 236.5  (199.2,281.4) | 3.1  (-1,7.4) |
| Republic of Côte d'Ivoire | 28617  (23715,34356) | 233.6  (195.7,277.4) | 62924  (52034,77221) | 230.5  (192.5,279.5) | -5.1  (-10.5,-0.6) |
| Republic of Cabo Verde | 940  (774,1140) | 253.4  (212.3,304.4) | 1396  (1159,1714) | 251.1  (208.2,308.1) | -5  (-8.9,-1.3) |
| Republic of the Gambia | 2096  (1712,2541) | 204.6  (168.5,246.1) | 4889  (3939,6001) | 209.7  (170.3,255.3) | 2.5  (-2.1,7.3) |
| Republic of Ghana | 31954  (26267,38890) | 211.8  (175.2,256.2) | 73207  (59530,90489) | 218.3  (178.6,266.8) | -2.8  (-7.6,1.9) |
| Republic of Guinea-Bissau | 2829  (2373,3384) | 271.3  (229.3,316.9) | 4772  (3985,5742) | 239.8  (203,287.3) | 5.2  (0.2,10.9) |
| Republic of Guinea | 15062  (12553,18188) | 231.7  (194.6,274) | 31233  (25945,37821) | 227.7  (191.4,273.4) | 6.6  (0.2,16.3) |
| Republic of Liberia | 14124  (8948,25833) | 503.7  (325.1,901.4) | 10754  (8709,13274) | 199.3  (162.6,244.3) | 6  (0,14.7) |
| Republic of Mali | 24745  (20508,29848) | 269.6  (225,324.5) | 63453  (52215,77229) | 255.9  (212.2,309.1) | **-60.4**  **(-77.7,-37.7)** |
| Islamic Republic of Mauritania | 5140  (4302,6208) | 248.2  (208.3,294.9) | 9559  (7860,11771) | 218.8  (182.8,267.2) | -15.7  (-19.5,-12.3) |
| Republic of the Niger | 21521  (17811,25575) | 249.8  (209.9,297.6) | 63614  (52114,78442) | 246.7  (204.8,300.2) | 21.3  (12.5,33) |
| Federal Republic of Nigeria | 222977  (182690,270432) | 237.4  (195.6,285.9) | 500436  (408101,614922) | 217.6  (178.4,264.4) | -11.6  (-14.8,-8.4) |
| Democratic Republic of Sao Tome and Principe | 373  (297,467) | 275.1  (222.3,336) | 722  (570,908) | 333.8  (265.1,421) | 1.8  (-1.9,5.4) |
| Togolese Republic | 9017  (7468,10912) | 236.8  (198.5,282.6) | 18365  (15159,22513) | 224.1  (186.7,273.8) | -1.3  (-4.8,2) |
| Republic of Senegal | 17590  (14375,21628) | 214.9  (177.1,258.1) | 32869  (26500,40595) | 208.8  (169.9,255.4) | 8.9  (4.2,13.7) |
| Republic of Sierra Leone | 10065  (8361,12095) | 219.3  (182.4,260.4) | 18953  (15473,23342) | 209.7  (171,255.7) | -3  (-17.2,7.4) |
| American Samoa | 151  (123,188) | 305.8  (249.4,376.4) | 145  (116,180) | 295.8  (238.8,370) | 1.1  (-5.2,7.1) |
| Greenland | 267  (201,349) | 496  (372.8,652.7) | 254  (187,345) | 418.1  (308.2,562.3) | -3.3  (-7.8,1.2) |
| Guam | 393  (312,498) | 275.9  (220.7,347.1) | 412  (327,516) | 266.1  (210.2,336.7) | -8.3  (-12,-5) |
| Bermuda | 181  (150,219) | 316.4  (259.6,384.8) | 186  (150,227) | 329.2  (263.9,402) | -2.9  (-8.4,2.9) |
| Principality of Monaco | 108  (77,145) | 409.2  (291.1,553.6) | 136  (97,183) | 416.7  (291.6,574.9) | 4.1  (-1.3,9.9) |
| Cook Islands | 62  (50,77) | 316.8  (255.3,389.7) | 53  (41,68) | 307.3  (236.5,396.9) | -5.3  (-8.3,-2.3) |
| Republic of Nauru | 32  (26,38) | 309.6  (256.7,374.4) | 38  (31,47) | 342.7  (280.8,424.9) | 1.1  (-3.2,4.7) |
| Puerto Rico | 14732  (12159,17693) | 406.6  (335.8,489.1) | 13955  (11197,17314) | 442.6  (356.1,549) | -1.2  (-5.4,3.1) |
| Republic of Niue | 6  (5,8) | 277.8  (226.9,340.8) | 5  (4,6) | 280.4  (224.8,354.4) | -24  (-36.9,-14) |
| Northern Mariana Islands | 193  (158,236) | 401.7  (328.2,491.7) | 183  (147,231) | 390.1  (312.4,493.9) | 0.9  (-4,6.1) |
| Saint Kitts and Nevis | 153  (126,187) | 363.2  (299.2,440.8) | 220  (179,270) | 395.9  (321.7,480.7) | -4.4  (-8,-0.8) |
| Tuvalu | 26  (22,31) | 279  (235.1,333.4) | 35  (28,43) | 278  (229.4,344.7) | -6  (-9.6,-2.5) |
| Republic of Palau | 68  (55,85) | 437.7  (355.8,544.8) | 84  (67,106) | 476.8  (378.7,610.7) | 9  (2.4,15.8) |
| United States Virgin Islands | 369  (310,437) | 347.9  (292.7,411) | 271  (225,326) | 327.1  (271.9,393.3) | -3.5  (-8.5,1.4) |
| Republic of San Marino | 94  (68,126) | 421.5  (302,573.3) | 126  (90,169) | 426.2  (301.8,587.5) | 8.9  (4.2,13.7) |
| Tokelau | 4  (3,5) | 255.6  (209.9,312.2) | 4  (3,5) | 258.5  (207.1,324.8) | 3.9  (-2.9,16.1) |
| Republic of South Sudan | 13601  (11313,16415) | 218.8  (183.6,259.3) | 22857  (19016,27992) | 227.4  (191.2,272.7) | 10.7  (5.4,15.8) |
| Republic of Sudan | 110281  (88839,140032) | 501.4  (408.5,626.1) | 177761  (144944,217670) | 381.3  (312.7,463.9) | -0.4  (-5.9,5.3) |

Age-standardized rates (ASRs) represent disease rates calculated per 100,000 population after adjusting for differences in age structure between populations. Percentage change in ASRs represents the relative change in age-standardized rates between 1990 and 2021, calculated as [(ASR₂₀₂₁ - ASR₁₉₉₀) / ASR₁₉₉₀] × 100%. The 95% UIs (uncertainty intervals) represent the range within which there is a 95% probability that the true value lies. Values in bold indicate regions experiencing substantial changes (>30%) during the study period.
