# Supplementary material for: The incidence, prevalence, and years lived with disability of forearm fractures: a systematic analysis based on the global burden of disease study 2021
Source: Front Public Health. 2025 Jul 10;13:1598660. doi: 10.3389/fpubh.2025.1598660 (PMC12287112; doi:10.3389/fpubh.2025.1598660)
Supplement: Supplementary file 2 [file Table_2.docx]

Supplementary Table 2. Years lived with disability (YLDs) cases of forearm fractures in 1990 and 2021 and the percentage change in the age-standardized rates (ASRs) per 100,000 population, for both sexes in 204 countries and territories.

| Country | 1990 | | 2021 | | Percentage change in  the ASRs per 100,000 |
| --- | --- | --- | --- | --- | --- |
|  | No  (95% UI) | ASRs per 100,000  (95% UI) | No  (95% UI) | ASRs per 100,000  (95% UI) |  |
| Global | 144166 (87129,229017) | 3 (1.8,4.7) | 205031 (126061,320235) | 2.5 (1.5,3.9) | -15.7 (-17.3,-14.1) |
| Taiwan (Province of China) | 501 (301,780) | 2.6 (1.6,4) | 453 (274,702) | 1.4 (0.8,2.2) | -21 (-28.5,-11.6) |
| People's Republic of China | 25546 (15320,40100) | 2.4 (1.5,3.8) | 42524 (26780,65689) | 2.5 (1.6,4) | 4.9 (0.6,8.7) |
| Kingdom of Cambodia | 198 (120,327) | 2.4 (1.5,3.8) | 375 (230,599) | 2.5 (1.6,4) | -24.9 (-28.1,-21.8) |
| Lao People's Democratic Republic | 69 (42,113) | 2 (1.2,3.2) | 103 (62,161) | 1.6 (1,2.4) | -1.1 (-10,8.9) |
| Democratic People's Republic of Korea | 332 (200,528) | 1.7 (1.1,2.8) | 411 (255,638) | 1.4 (0.8,2.1) | 7 (-7.3,18.9) |
| Malaysia | 249 (149,402) | 1.7 (1,2.7) | 520 (310,816) | 1.7 (1,2.6) | -19.9 (-38.1,-3.2) |
| Democratic Socialist Republic of Sri Lanka | 457 (260,773) | 2.8 (1.6,4.7) | 611 (390,952) | 2.6 (1.6,4) | -0.4 (-10.5,10) |
| Republic of Maldives | 3 (2,6) | 1.9 (1.1,3) | 9 (5,14) | 1.8 (1.1,2.8) | -14.2 (-23.5,-3.9) |
| Republic of Indonesia | 3499 (2103,5615) | 2.3 (1.4,3.6) | 4364 (2677,6863) | 1.7 (1.1,2.7) | -6.6 (-15.9,2.5) |
| Republic of the Union of Myanmar | 831 (487,1362) | 2.3 (1.4,3.7) | 1240 (776,1975) | 2.3 (1.5,3.7) | -8.1 (-18.4,1.8) |
| Republic of the Philippines | 1147 (677,1845) | 2.1 (1.3,3.4) | 1658 (999,2615) | 1.6 (1,2.5) | 5.3 (-1.6,12.6) |
| Kingdom of Thailand | 1245 (743,1960) | 2.4 (1.5,3.7) | 1742 (1090,2703) | 2.1 (1.3,3.4) | -11 (-18.3,-3.2) |
| Republic of Fiji | 10 (6,15) | 1.5 (0.9,2.3) | 12 (7,18) | 1.4 (0.8,2.1) | -27.5 (-34.2,-21) |
| Democratic Republic of Timor-Leste | 16 (9,27) | 2.2 (1.3,3.7) | 20 (13,33) | 1.8 (1.1,2.8) | -10.2 (-17.1,-3.2) |
| Socialist Republic of Viet Nam | 1128 (675,1821) | 2.1 (1.2,3.2) | 2304 (1427,3698) | 2.4 (1.5,3.8) | 24.2 (14.3,36.5) |
| Republic of Kiribati | 1 (1,1) | 1.3 (0.8,2.1) | 1 (1,2) | 1.1 (0.7,1.8) | -0.6 (-9.8,10.2) |
| Independent State of Papua New Guinea | 62 (37,98) | 2 (1.3,3.1) | 201 (120,315) | 2.5 (1.5,3.9) | **-46.1 (-50.9,-40.8)** |
| Republic of the Marshall Islands | 1 (0,1) | 1.8 (1.1,2.7) | 1 (1,1) | 1.7 (1.1,2.7) | -9 (-29.1,7.7) |
| Federated States of Micronesia | 1 (1,2) | 1.8 (1.1,2.8) | 2 (1,3) | 1.9 (1.2,3) | -1.7 (-10.1,7.1) |
| Independent State of Samoa | 2 (1,4) | 1.8 (1.1,2.8) | 3 (2,5) | 1.8 (1.1,2.7) | -19 (-26,-12) |
| Solomon Islands | 6 (4,10) | 2.8 (1.7,4.3) | 17 (10,27) | 3.4 (2.1,5.2) | -20.1 (-28.9,-11.4) |
| Republic of Vanuatu | 2 (1,3) | 1.5 (0.9,2.4) | 4 (2,6) | 1.5 (0.9,2.4) | 11.5 (3.2,19.7) |
| Kingdom of Tonga | 1 (1,2) | 1.5 (0.9,2.3) | 1 (1,2) | 1.3 (0.8,2.1) | **-46.1 (-51.2,-40.8)** |
| Republic of Armenia | 144 (88,228) | 4.4 (2.7,6.9) | 76 (47,118) | 2.3 (1.4,3.7) | 4.3 (-5,14.8) |
| Georgia | 242 (144,382) | 4.3 (2.6,6.8) | 178 (107,282) | 4.5 (2.7,7.3) | -13.1 (-21.7,-3.7) |
| Republic of Kazakhstan | 671 (398,1061) | 4.2 (2.5,6.6) | 689 (418,1064) | 3.7 (2.2,5.7) | **-32.7 (-37,-28.8)** |
| Republic of Azerbaijan | 202 (118,324) | 2.8 (1.7,4.5) | 244 (149,385) | 2.3 (1.4,3.7) | -25.1 (-28.5,-21.6) |
| Kyrgyz Republic | 161 (96,258) | 3.8 (2.3,6) | 162 (96,259) | 2.5 (1.5,3.9) | -2.6 (-11.1,7.5) |
| Mongolia | 67 (39,109) | 3.5 (2.1,5.6) | 125 (75,200) | 3.9 (2.4,6.2) | **-35.3 (-39.9,-29.7)** |
| Turkmenistan | 103 (60,170) | 2.9 (1.8,4.7) | 116 (67,191) | 2.3 (1.3,3.7) | -18.6 (-24.8,-12.6) |
| Bosnia and Herzegovina | 233 (139,380) | 5.2 (3.1,8.5) | 161 (100,256) | 4.4 (2.6,7.1) | 15.6 (5.9,26.3) |
| Republic of Tajikistan | 159 (93,259) | 3.2 (1.9,5.1) | 223 (134,357) | 2.3 (1.4,3.7) | -23.8 (-26.3,-21.5) |
| Republic of Uzbekistan | 614 (360,994) | 3.1 (1.9,4.9) | 834 (503,1347) | 2.5 (1.5,4) | -15.7 (-21.4,-9.6) |
| Republic of Albania | 166 (97,268) | 5.2 (3.1,8.4) | 134 (81,214) | 4.7 (2.8,7.6) | 20.1 (10.1,30.4) |
| Republic of Bulgaria | 590 (358,939) | 6.5 (3.9,10.4) | 408 (251,633) | 5.3 (3.1,8.4) | -18.5 (-23.6,-13.6) |
| North Macedonia | 93 (55,149) | 4.7 (2.8,7.5) | 100 (61,158) | 4.3 (2.6,6.9) | -22.9 (-26.9,-18.5) |
| Republic of Croatia | 339 (206,539) | 6.6 (4,10.7) | 336 (209,511) | 5.6 (3.4,8.8) | -21.6 (-27.7,-14.5) |
| Czech Republic | 902 (551,1400) | 7.9 (4.8,12.5) | 708 (436,1101) | 5.3 (3.2,8.5) | -15.1 (-20.1,-9.8) |
| Hungary | 917 (568,1428) | 7.7 (4.7,12) | 646 (403,1018) | 5.1 (3.1,8.2) | **-42.5 (-46.2,-38.9)** |
| Montenegro | 36 (21,58) | 5.7 (3.4,9.2) | 33 (20,52) | 4.8 (2.9,7.8) | -13.1 (-19,-7) |
| Republic of Serbia | 490 (298,778) | 5 (3,7.9) | 451 (274,709) | 4.4 (2.6,7) | -22.1 (-26.8,-16.6) |
| Slovak Republic | 391 (240,630) | 7.1 (4.4,11.5) | 380 (235,602) | 5.8 (3.5,9.4) | -3.9 (-10.7,2.9) |
| Republic of Poland | 2461 (1483,3938) | 6.2 (3.7,10) | 2242 (1381,3514) | 4.7 (2.8,7.6) | -16 (-22.6,-8.5) |
| Republic of Slovenia | 183 (113,286) | 8.5 (5.2,13.5) | 188 (117,289) | 6.6 (4,10.5) | -26.7 (-29.2,-24.3) |
| Romania | 1672 (1007,2677) | 6.9 (4.1,11.1) | 1120 (695,1741) | 5.1 (3,8.1) | -2.8 (-9.1,3.3) |
| Republic of Estonia | 120 (74,189) | 7.1 (4.3,11.3) | 67 (41,105) | 4.3 (2.5,6.8) | -7.6 (-15.2,-0.1) |
| Republic of Belarus | 629 (382,1002) | 5.7 (3.5,9.2) | 607 (376,933) | 5.6 (3.4,8.8) | -17.9 (-22.1,-13.8) |
| Republic of Latvia | 234 (142,366) | 8 (4.8,12.7) | 107 (68,165) | 4.6 (2.8,7.4) | **-40 (-44.1,-35.9)** |
| Russian Federation | 10331 (6229,16580) | 6.5 (3.9,10.5) | 8807 (5345,13737) | 5.3 (3.1,8.3) | -15.8 (-23.1,-8.4) |
| Republic of Lithuania | 279 (172,446) | 7.1 (4.3,11.4) | 178 (110,273) | 5 (3,7.8) | -19.2 (-22.1,-16.5) |
| Ukraine | 3868 (2326,6262) | 6.8 (4.1,11.2) | 2672 (1618,4156) | 5.3 (3.2,8.5) | -21.4 (-26.9,-15.4) |
| Japan | 3064 (1872,4836) | 2.2 (1.3,3.6) | 3034 (1898,4634) | 1.6 (1,2.6) | 6.5 (-1.2,14.2) |
| Republic of Moldova | 263 (159,421) | 5.9 (3.6,9.5) | 150 (93,236) | 3.6 (2.2,5.8) | -11.1 (-17.3,-4.8) |
| Republic of Korea | 1481 (894,2346) | 3.6 (2.2,5.6) | 1675 (1055,2624) | 2.5 (1.5,4) | -10.5 (-18.8,-2.8) |
| Brunei Darussalam | 6 (3,9) | 2.5 (1.5,3.9) | 9 (5,15) | 2.1 (1.3,3.3) | -12.9 (-18.6,-6.7) |
| Republic of Singapore | 63 (38,102) | 2.1 (1.3,3.3) | 118 (71,187) | 1.9 (1.1,3) | **-33.2 (-38,-28.9)** |
| New Zealand | 145 (89,229) | 4.1 (2.5,6.5) | 211 (128,328) | 3.7 (2.2,5.8) | -20.7 (-27.9,-14.8) |
| Australia | 588 (357,935) | 3.4 (2.1,5.4) | 1011 (618,1573) | 3.3 (1.9,5.3) | **-38.3 (-42.5,-34)** |
| Principality of Andorra | 2 (1,3) | 3.9 (2.3,6.1) | 5 (3,7) | 4.1 (2.5,6.5) | -14.7 (-21.3,-7.1) |
| Kingdom of Belgium | 422 (257,656) | 3.6 (2.2,5.8) | 602 (375,919) | 3.7 (2.2,5.8) | -9.7 (-17.9,-1.1) |
| Republic of Cyprus | 24 (15,38) | 3.2 (1.9,4.9) | 45 (28,70) | 2.9 (1.8,4.6) | -26.8 (-31.2,-22.2) |
| Republic of Austria | 350 (213,552) | 3.9 (2.3,6.1) | 360 (223,557) | 3 (1.8,4.7) | **-35 (-40.4,-29.2)** |
| Kingdom of Denmark | 202 (125,314) | 3.2 (1.9,5) | 186 (114,289) | 2.5 (1.5,4.1) | -26.3 (-32.6,-19.4) |
| Republic of Finland | 243 (148,381) | 4.3 (2.6,6.9) | 294 (181,453) | 3.8 (2.3,6.1) | -7.7 (-14.6,-0.3) |
| Federal Republic of Germany | 2861 (1750,4493) | 3 (1.8,4.9) | 3284 (2022,5014) | 2.7 (1.6,4.3) | -29.7 (-33.7,-25.4) |
| French Republic | 2520 (1509,3917) | 3.8 (2.3,5.9) | 3128 (1946,4767) | 3.3 (2,5.3) | -22.1 (-25.9,-18.3) |
| Hellenic Republic | 370 (226,576) | 3.2 (1.9,5.1) | 297 (184,457) | 2.4 (1.4,3.8) | -5.5 (-8.1,-3.1) |
| Republic of Iceland | 8 (5,12) | 3 (1.8,4.7) | 11 (6,17) | 2.7 (1.6,4.4) | -10.2 (-17.3,-2.2) |
| State of Israel | 113 (67,180) | 2.3 (1.4,3.6) | 223 (136,356) | 2.2 (1.3,3.6) | -29.9 (-36.6,-23.7) |
| Ireland | 101 (59,159) | 2.7 (1.6,4.3) | 149 (91,234) | 2.7 (1.6,4.3) | -17.9 (-21.7,-14.1) |
| Republic of Italy | 2533 (1562,3987) | 3.8 (2.3,6) | 2392 (1519,3658) | 2.9 (1.8,4.7) | -4.8 (-12.7,4.1) |
| Grand Duchy of Luxembourg | 16 (10,24) | 3.6 (2.2,5.7) | 24 (15,38) | 3.1 (1.9,4.9) | 1.5 (-5.3,8.9) |
| Kingdom of Norway | 167 (103,263) | 3.2 (1.9,5) | 192 (120,302) | 2.6 (1.6,4.1) | -10.3 (-17.2,-3.7) |
| Republic of Malta | 12 (7,18) | 3.1 (1.8,4.8) | 16 (10,24) | 2.9 (1.7,4.5) | -3.6 (-12.2,6.1) |
| Portuguese Republic | 328 (201,520) | 3 (1.8,4.8) | 287 (180,440) | 1.9 (1.2,3) | -11.6 (-18,-5.5) |
| Kingdom of the Netherlands | 358 (222,563) | 2.2 (1.3,3.5) | 612 (383,950) | 2.4 (1.5,3.9) | -24.5 (-30,-18.7) |
| Kingdom of Spain | 1060 (643,1695) | 2.5 (1.5,4) | 1491 (931,2331) | 2.6 (1.5,4.1) | -4.5 (-13,3.9) |
| Swiss Confederation | 375 (232,577) | 4.6 (2.8,7.3) | 417 (260,650) | 3.5 (2.1,5.5) | -6.3 (-14.3,4.4) |
| Kingdom of Sweden | 309 (187,483) | 2.9 (1.8,4.6) | 346 (217,535) | 2.4 (1.4,3.8) | 16.1 (5.6,27.3) |
| United Kingdom of Great Britain and Northern Ireland | 1615 (988,2520) | 2.5 (1.5,3.9) | 2033 (1268,3135) | 2.3 (1.4,3.7) | 12 (1.6,22.1) |
| Argentine Republic | 642 (379,1025) | 2 (1.2,3.1) | 901 (542,1471) | 1.9 (1.1,3.1) | -2.6 (-10.9,5.3) |
| Eastern Republic of Uruguay | 78 (47,125) | 2.4 (1.4,3.8) | 89 (55,139) | 2.2 (1.4,3.6) | 5.7 (-2.7,14.1) |
| Republic of Chile | 277 (166,438) | 2.2 (1.3,3.5) | 521 (320,811) | 2.6 (1.5,4.1) | -9 (-13.4,-3.7) |
| Canada | 622 (382,990) | 2.1 (1.3,3.3) | 1110 (691,1732) | 1.9 (1.2,3) | 3.5 (-5.6,13.7) |
| United States of America | 6493 (4018,10236) | 2.3 (1.4,3.7) | 9990 (6233,15481) | 2.1 (1.3,3.4) | 4.5 (-4.9,13.7) |
| Antigua and Barbuda | 1 (1,2) | 2 (1.2,3.2) | 2 (1,3) | 2.1 (1.3,3.3) | -7 (-13.9,0.3) |
| Commonwealth of the Bahamas | 4 (3,7) | 1.8 (1.1,2.9) | 8 (5,12) | 1.9 (1.2,3) | -17.1 (-23,-11.5) |
| Belize | 3 (2,5) | 1.9 (1.1,3) | 9 (5,14) | 2.1 (1.3,3.5) | 5.2 (-5.2,14.2) |
| Barbados | 4 (3,7) | 1.6 (1,2.6) | 6 (4,9) | 1.7 (1,2.7) | 18.2 (8.4,29.4) |
| Republic of Cuba | 290 (174,464) | 2.7 (1.6,4.3) | 467 (291,715) | 3.1 (1.9,4.8) | -0.1 (-9.1,8.9) |
| Commonwealth of Dominica | 1 (1,2) | 1.6 (1,2.6) | 1 (1,2) | 1.7 (1,2.7) | -3 (-11.9,5.8) |
| Dominican Republic | 108 (64,177) | 1.7 (1,2.7) | 218 (131,350) | 2 (1.2,3.2) | -7.9 (-19.1,2) |
| Republic of Guyana | 15 (9,24) | 2.2 (1.3,3.5) | 18 (11,28) | 2.5 (1.5,3.8) | -23.2 (-25.5,-20.3) |
| Grenada | 2 (1,3) | 1.9 (1.2,3.1) | 2 (1,3) | 2.1 (1.2,3.3) | -18.6 (-27.3,-10.4) |
| Republic of Haiti | 124 (75,200) | 2.2 (1.4,3.5) | 301 (185,474) | 2.5 (1.6,3.9) | -13.9 (-22.6,-5.8) |
| Jamaica | 42 (25,68) | 1.8 (1.1,3) | 54 (32,86) | 1.8 (1.1,3) | -11 (-18.1,-3.6) |
| Saint Vincent and the Grenadines | 2 (1,3) | 1.8 (1.1,2.8) | 2 (1,4) | 2 (1.2,3.2) | 5 (-4.7,14.6) |
| Saint Lucia | 2 (1,4) | 1.8 (1.1,2.8) | 4 (2,6) | 1.8 (1.1,2.9) | **-32.5 (-37.8,-26.8)** |
| Republic of Suriname | 6 (4,10) | 1.7 (1.1,2.8) | 11 (7,17) | 1.8 (1.1,2.9) | 4.5 (-5,14.5) |
| Republic of Trinidad and Tobago | 21 (13,34) | 1.9 (1.2,3) | 27 (17,43) | 1.8 (1.1,3) | 1.7 (-6.3,9.8) |
| Republic of Ecuador | 225 (134,362) | 2.6 (1.6,4) | 463 (281,732) | 2.6 (1.6,4.1) | 14.9 (-1.3,42.7) |
| Plurinational State of Bolivia | 134 (80,219) | 2.4 (1.5,3.9) | 239 (143,380) | 2.2 (1.3,3.4) | -9.4 (-15.9,-2.5) |
| Republic of Peru | 458 (269,765) | 2.3 (1.4,3.7) | 760 (454,1218) | 2.1 (1.3,3.4) | **-36.7 (-38.6,-34.8)** |
| Republic of Costa Rica | 82 (49,135) | 3 (1.8,4.8) | 133 (81,212) | 2.7 (1.6,4.4) | 10.2 (0.4,20.4) |
| Republic of El Salvador | 185 (113,306) | 3.7 (2.3,6) | 194 (118,301) | 3 (1.8,4.6) | 12.9 (4.1,22.1) |
| Republic of Colombia | 1104 (653,1807) | 3.7 (2.3,5.9) | 1249 (766,1987) | 2.5 (1.5,4.1) | 12.7 (4,21.1) |
| Republic of Guatemala | 261 (154,434) | 3.6 (2.2,5.7) | 486 (289,792) | 3.3 (2,5.3) | -10.8 (-17.4,-2.3) |
| Republic of Honduras | 123 (72,204) | 2.8 (1.7,4.5) | 232 (141,377) | 2.5 (1.6,4) | -12.4 (-18.8,-5.7) |
| Republic of Nicaragua | 102 (63,167) | 2.8 (1.8,4.5) | 148 (91,238) | 2.4 (1.5,3.8) | 15 (6.4,24.7) |
| United Mexican States | 3604 (2110,5850) | 4.8 (2.9,7.6) | 3908 (2357,6138) | 3 (1.8,4.8) | 5.7 (-3.7,15.2) |
| Republic of Panama | 62 (36,103) | 2.7 (1.6,4.5) | 99 (60,161) | 2.3 (1.4,3.7) | 2.7 (-6.4,13.2) |
| Bolivarian Republic of Venezuela | 597 (357,980) | 3.4 (2.1,5.6) | 810 (494,1295) | 3 (1.8,4.8) | -6.6 (-15.5,2) |
| Federative Republic of Brazil | 6025 (3560,9464) | 4.4 (2.7,6.9) | 7894 (4750,12226) | 3.4 (2,5.3) | -16.6 (-23.5,-9.7) |
| Republic of Paraguay | 113 (67,184) | 3 (1.8,4.8) | 194 (114,309) | 2.8 (1.6,4.4) | -8.9 (-16.6,-0.5) |
| Kingdom of Bahrain | 10 (6,16) | 2.1 (1.3,3.4) | 30 (18,48) | 2 (1.2,3.1) | -25.2 (-31.6,-18.5) |
| People's Democratic Republic of Algeria | 721 (423,1149) | 3.2 (2,5.1) | 1031 (616,1598) | 2.4 (1.4,3.7) | -23.4 (-28.8,-16.2) |
| Arab Republic of Egypt | 1343 (800,2210) | 2.7 (1.6,4.3) | 2023 (1216,3187) | 2.1 (1.3,3.3) | 8.6 (-1.3,20.2) |
| Islamic Republic of Iran | 1956 (1165,3211) | 3.9 (2.3,6.1) | 2125 (1300,3292) | 2.5 (1.5,3.8) | -11.5 (-19.2,-4) |
| Republic of Iraq | 652 (416,1050) | 4 (2.6,6.3) | 1164 (745,1873) | 3 (2,4.8) | -3.1 (-11.5,6.3) |
| State of Kuwait | 55 (30,93) | 3.3 (1.8,5.5) | 116 (71,185) | 2.4 (1.5,3.8) | **-35.8 (-43.2,-30.6)** |
| Hashemite Kingdom of Jordan | 77 (44,123) | 2.4 (1.4,3.7) | 224 (129,358) | 1.9 (1.1,3) | **-38 (-47.7,-27.1)** |
| Lebanese Republic | 94 (58,155) | 3.5 (2.2,5.6) | 125 (78,197) | 2.2 (1.3,3.4) | -23.3 (-29.7,-17.2) |
| Palestine | 51 (32,84) | 2.8 (1.8,4.4) | 118 (73,189) | 2.6 (1.6,4) | -20.3 (-26.6,-12.9) |
| State of Libya | 100 (59,162) | 2.7 (1.7,4.4) | 194 (118,303) | 3 (1.8,4.6) | -11.6 (-18.5,-4.6) |
| Kingdom of Morocco | 703 (417,1152) | 3.1 (1.8,5) | 975 (578,1564) | 2.7 (1.6,4.3) | -8.1 (-10.6,-5.4) |
| Sultanate of Oman | 65 (39,106) | 4.2 (2.5,6.5) | 130 (78,210) | 3.2 (1.9,5.1) | -27.2 (-42.1,-15.1) |
| State of Qatar | 14 (8,22) | 3.2 (1.9,5) | 85 (50,137) | 2.8 (1.7,4.5) | -8.4 (-18.1,2.2) |
| Kingdom of Saudi Arabia | 720 (424,1164) | 5.6 (3.4,9) | 2337 (1389,3672) | 6.1 (3.7,9.6) | **32.6 (20.2,45.7)** |
| Republic of Tunisia | 212 (128,336) | 2.8 (1.7,4.3) | 299 (184,466) | 2.4 (1.5,3.8) | 23.7 (7.7,53) |
| Syrian Arab Republic | 257 (147,415) | 2.3 (1.3,3.6) | 387 (242,622) | 2.8 (1.8,4.5) | -7.1 (-16,1.6) |
| Republic of Turkey | 1215 (720,1986) | 2.3 (1.4,3.6) | 1878 (1160,2862) | 2.2 (1.3,3.4) | 8.3 (-7.4,33.9) |
| United Arab Emirates | 55 (33,87) | 3.3 (2,5.2) | 315 (189,487) | 3 (1.8,4.7) | 7.4 (-14.4,42.3) |
| Republic of Yemen | 297 (177,494) | 2.7 (1.6,4.3) | 881 (503,1495) | 2.9 (1.7,4.8) | -19.4 (-27.3,-12.2) |
| Islamic Republic of Afghanistan | 299 (179,518) | 3.4 (2,5.9) | 1000 (553,1772) | 3.6 (2.1,6.2) | -0.7 (-11,11) |
| Republic of India | 21600 (12697,34390) | 3.3 (2,5.2) | 38458 (23352,58666) | 3 (1.9,4.6) | -13.2 (-20.6,-6.2) |
| People's Republic of Bangladesh | 1223 (702,1955) | 1.3 (0.7,2) | 2133 (1268,3381) | 1.3 (0.8,2.1) | 10.2 (3.5,17.4) |
| Kingdom of Bhutan | 9 (5,14) | 1.7 (1,2.7) | 16 (9,25) | 2.3 (1.4,3.6) | 4.7 (-5.2,16.5) |
| Federal Democratic Republic of Nepal | 442 (258,696) | 2.8 (1.7,4.3) | 897 (541,1389) | 3.1 (1.9,4.8) | 8 (-11,49) |
| Islamic Republic of Pakistan | 1351 (794,2176) | 1.4 (0.9,2.3) | 2617 (1580,4170) | 1.3 (0.8,2) | -9.1 (-16,-1) |
| Republic of Angola | 165 (98,281) | 1.9 (1.1,3) | 360 (220,584) | 1.5 (0.9,2.3) | -10.3 (-17,-3.2) |
| Central African Republic | 33 (20,55) | 1.6 (0.9,2.5) | 79 (45,130) | 1.7 (1,2.8) | -10.2 (-19.5,2.8) |
| Democratic Republic of the Congo | 447 (263,745) | 1.5 (0.9,2.3) | 1003 (600,1628) | 1.5 (0.9,2.3) | -20 (-31.6,-8.6) |
| Republic of Equatorial Guinea | 5 (3,8) | 1.5 (0.9,2.3) | 14 (8,22) | 1.3 (0.8,2) | **-45.1 (-56.7,-35.4)** |
| Republic of the Congo | 28 (17,45) | 1.5 (0.9,2.4) | 60 (37,96) | 1.4 (0.9,2.2) | 10.9 (-3.2,30.4) |
| Republic of Burundi | 65 (38,105) | 1.5 (0.9,2.3) | 160 (101,256) | 1.6 (1,2.5) | -1 (-10.6,9.1) |
| Gabonese Republic | 13 (8,21) | 1.6 (1,2.5) | 21 (13,34) | 1.5 (0.9,2.3) | 12.7 (3.4,22) |
| Republic of Djibouti | 5 (3,8) | 1.4 (0.9,2.3) | 13 (8,21) | 1.3 (0.8,2.1) | **-49.1 (-67.3,-27.8)** |
| State of Eritrea | 119 (57,230) | 3.5 (1.8,6.4) | 86 (54,140) | 1.8 (1.2,2.9) | -9.6 (-22.6,3.8) |
| Union of the Comoros | 5 (3,8) | 1.4 (0.9,2.2) | 8 (5,13) | 1.3 (0.8,2) | -10.4 (-19.4,-1.9) |
| Federal Democratic Republic of Ethiopia | 1130 (604,1963) | 2.6 (1.5,4.2) | 1195 (691,1956) | 1.4 (0.9,2.3) | -4.6 (-13.5,6.2) |
| Republic of Madagascar | 128 (75,206) | 1.3 (0.8,2) | 248 (144,396) | 1.1 (0.7,1.7) | -11.5 (-19.7,-1.1) |
| Republic of Kenya | 238 (141,385) | 1.4 (0.9,2.2) | 506 (301,803) | 1.4 (0.8,2.1) | -14.2 (-22.5,-4) |
| Republic of Malawi | 102 (60,165) | 1.3 (0.8,2) | 180 (106,283) | 1.2 (0.7,1.9) | **-36.1 (-39.3,-33)** |
| Republic of Mauritius | 15 (9,23) | 1.4 (0.9,2.3) | 20 (12,33) | 1.4 (0.9,2.3) | -8.3 (-18.4,1.7) |
| Republic of Mozambique | 190 (114,313) | 1.7 (1,2.7) | 345 (209,561) | 1.5 (1,2.4) | -2.1 (-10.5,7.2) |
| Republic of Seychelles | 1 (1,2) | 1.7 (1,2.7) | 2 (1,3) | 1.5 (0.9,2.4) | 2.4 (-7,12.1) |
| Republic of Rwanda | 112 (62,191) | 1.8 (1.1,3) | 169 (105,272) | 1.7 (1,2.6) | -14.3 (-22.2,-5.5) |
| Federal Republic of Somalia | 113 (64,195) | 1.7 (1,2.9) | 253 (152,422) | 1.6 (1,2.4) | -10.3 (-31.4,25.5) |
| United Republic of Tanzania | 291 (171,474) | 1.4 (0.8,2.2) | 623 (364,1002) | 1.3 (0.8,2.1) | -3.9 (-14.4,7.8) |
| Republic of Zambia | 86 (51,144) | 1.4 (0.9,2.2) | 202 (120,319) | 1.4 (0.9,2.2) | -6.6 (-15.7,2.8) |
| Republic of Uganda | 202 (124,330) | 1.4 (0.9,2.3) | 433 (266,699) | 1.4 (0.9,2.1) | 9.1 (-1.2,20.1) |
| Republic of Botswana | 16 (10,26) | 1.5 (0.9,2.4) | 36 (22,57) | 1.6 (1,2.6) | -1.1 (-10.6,9.5) |
| Kingdom of Lesotho | 19 (11,31) | 1.5 (0.9,2.3) | 30 (18,49) | 1.9 (1.1,2.9) | 0.9 (-8.6,11.6) |
| Republic of South Africa | 826 (501,1318) | 2.6 (1.6,4) | 931 (563,1463) | 1.7 (1,2.6) | -5 (-15.3,7.7) |
| Republic of Namibia | 18 (11,29) | 1.6 (1,2.5) | 32 (19,51) | 1.5 (0.9,2.4) | -8.1 (-20.4,6) |
| Kingdom of Eswatini | 10 (6,16) | 1.7 (1,2.6) | 17 (10,27) | 1.7 (1,2.7) | -2.9 (-5.6,0) |
| Republic of Zimbabwe | 112 (67,182) | 1.5 (0.9,2.3) | 164 (98,264) | 1.4 (0.9,2.1) | 26.1 (13.9,41.6) |
| Burkina Faso | 115 (68,187) | 1.5 (0.9,2.4) | 280 (163,450) | 1.6 (1,2.5) | 0.1 (-9.4,11.3) |
| Republic of Benin | 56 (33,95) | 1.4 (0.9,2.3) | 146 (84,236) | 1.4 (0.8,2.2) | -2.7 (-11.5,6.6) |
| Republic of Cameroon | 111 (66,179) | 1.4 (0.9,2.2) | 348 (207,571) | 1.4 (0.9,2.3) | 3.8 (-6.2,13.4) |
| Republic of Cabo Verde | 5 (3,8) | 1.5 (0.9,2.4) | 8 (5,13) | 1.5 (0.9,2.4) | -6.6 (-15,2.7) |
| Republic of Côte d'Ivoire | 130 (78,215) | 1.4 (0.9,2.3) | 303 (175,487) | 1.4 (0.9,2.2) | -13.7 (-21.3,-5.5) |
| Republic of the Gambia | 10 (6,16) | 1.3 (0.8,2) | 23 (14,38) | 1.3 (0.8,2.1) | 3.8 (-6,13.6) |
| Republic of Chad | 75 (45,124) | 1.5 (0.9,2.3) | 195 (113,326) | 1.4 (0.9,2.3) | 0.4 (-8.4,10.6) |
| Republic of Ghana | 152 (89,249) | 1.3 (0.8,2.1) | 368 (221,594) | 1.4 (0.8,2.2) | 5.1 (-4.6,14.6) |
| Republic of Guinea | 73 (44,119) | 1.4 (0.9,2.3) | 149 (88,239) | 1.4 (0.9,2.2) | 1.1 (-9.5,12) |
| Republic of Liberia | 54 (27,102) | 2.2 (1.2,3.9) | 55 (34,90) | 1.3 (0.8,2.1) | 2.6 (-6,11.7) |
| Republic of Guinea-Bissau | 13 (8,22) | 1.7 (1.1,2.7) | 23 (14,38) | 1.6 (0.9,2.4) | 21 (10,33.1) |
| Republic of Mali | 115 (67,191) | 1.6 (1,2.6) | 294 (172,484) | 1.6 (1,2.5) | -1.5 (-10.1,8.1) |
| Islamic Republic of Mauritania | 26 (15,42) | 1.6 (1,2.6) | 48 (29,77) | 1.4 (0.9,2.2) | 0.1 (-8.6,9.4) |
| Federal Republic of Nigeria | 1086 (648,1759) | 1.5 (0.9,2.3) | 2398 (1416,3859) | 1.4 (0.8,2.1) | **-40.5 (-62.2,-18.8)** |
| Republic of the Niger | 97 (58,160) | 1.6 (1,2.4) | 283 (164,470) | 1.5 (0.9,2.4) | -6.3 (-9.3,-2.8) |
| Democratic Republic of Sao Tome and Principe | 2 (1,3) | 1.6 (1,2.6) | 3 (2,6) | 1.9 (1.2,3) | -3.3 (-11.7,5.8) |
| Republic of Senegal | 80 (47,133) | 1.3 (0.8,2) | 162 (98,261) | 1.3 (0.8,2) | -9.3 (-17.3,0.5) |
| Republic of Sierra Leone | 48 (28,79) | 1.3 (0.8,2.1) | 95 (57,155) | 1.4 (0.8,2.2) | 2.5 (-7.6,16.3) |
| American Samoa | 1 (0,1) | 1.8 (1.1,2.8) | 1 (1,1) | 1.8 (1.1,2.7) | -1.4 (-9.6,7.6) |
| Togolese Republic | 41 (25,67) | 1.5 (0.9,2.3) | 93 (56,146) | 1.4 (0.9,2.2) | -2.6 (-10.9,6.7) |
| Bermuda | 1 (1,2) | 1.8 (1.1,2.9) | 1 (1,2) | 1.8 (1.1,2.9) | 1 (-7.4,9) |
| Cook Islands | 0 (0,1) | 1.9 (1.1,3) | 0 (0,1) | 1.8 (1.1,2.9) | -18.7 (-24.4,-12.9) |
| Guam | 2 (1,3) | 1.7 (1,2.7) | 3 (2,4) | 1.6 (1,2.6) | -1.4 (-10.4,9) |
| Greenland | 2 (1,2) | 3.5 (2.1,5.5) | 2 (1,3) | 2.8 (1.7,4.4) | 4.1 (-4.8,12.7) |
| Republic of Nauru | 0 (0,0) | 2 (1.2,3.1) | 0 (0,0) | 2.2 (1.3,3.4) | -2.4 (-13.1,7.8) |
| Principality of Monaco | 1 (1,1) | 2.4 (1.4,3.8) | 1 (1,2) | 2.4 (1.4,3.9) | 0.6 (-7.7,9.7) |
| Republic of Niue | 0 (0,0) | 1.7 (1.1,2.7) | 0 (0,0) | 1.7 (1,2.6) | -2 (-10,7.8) |
| Northern Mariana Islands | 1 (1,1) | 2.4 (1.4,3.8) | 1 (1,2) | 2.4 (1.4,3.7) | -18.5 (-27.1,-9.2) |
| Saint Kitts and Nevis | 1 (0,1) | 2.1 (1.3,3.3) | 1 (1,2) | 2.2 (1.3,3.5) | -7.2 (-15.2,0.5) |
| Republic of Palau | 0 (0,1) | 2.8 (1.7,4.3) | 1 (0,1) | 2.9 (1.8,4.6) | 4.4 (-7.3,18.2) |
| Republic of San Marino | 1 (0,1) | 2.5 (1.5,4) | 1 (1,2) | 2.5 (1.5,4) | -1.6 (-11,10.6) |
| Puerto Rico | 85 (51,139) | 2.4 (1.4,3.9) | 110 (68,171) | 2.5 (1.5,4) | -0.4 (-9.1,8.3) |
| Tokelau | 0 (0,0) | 1.6 (1,2.6) | 0 (0,0) | 1.6 (1,2.5) | 4.4 (-4.7,13.3) |
| United States Virgin Islands | 2 (1,3) | 2 (1.2,3.3) | 2 (1,3) | 1.9 (1.1,3) | 6.4 (-1.7,15.3) |
| Tuvalu | 0 (0,0) | 1.8 (1.1,2.9) | 0 (0,0) | 1.8 (1.1,2.8) | -4.2 (-13.7,7.5) |
| Republic of Sudan | 489 (288,824) | 2.7 (1.6,4.4) | 847 (510,1345) | 2.2 (1.4,3.5) | -3 (-13.7,8.7) |
| Republic of South Sudan | 65 (39,105) | 1.3 (0.8,2.1) | 109 (67,176) | 1.4 (0.9,2.1) | 10.6 (0.4,21.3) |

Age-standardized rates (ASRs) represent disease rates calculated per 100,000 population after adjusting for differences in age structure between populations. Percentage change in ASRs represents the relative change in age-standardized rates between 1990 and 2021, calculated as [(ASR₂₀₂₁ - ASR₁₉₉₀) / ASR₁₉₉₀] × 100%. The 95% UIs (uncertainty intervals) represent the range within which there is a 95% probability that the true value lies. Values in bold indicate regions experiencing substantial changes (>30%) during the study period.
