# Supplementary material for: The incidence, prevalence, and years lived with disability of forearm fractures: a systematic analysis based on the global burden of disease study 2021
Source: Front Public Health. 2025 Jul 10;13:1598660. doi: 10.3389/fpubh.2025.1598660 (PMC12287112; doi:10.3389/fpubh.2025.1598660)
Supplement: Supplementary file 3 [file Data_Sheet_1.docx]

1. R code for Table:

setwd("D:/GBD/table4s")

```{R}

library(tidyverse)

```

```{R}

Global_21Regions_204counties_1990to2021 <- read.csv('D:/GBD/table4s/Global_21Regions_204counties_1990to2021.csv', header=T)

Global_21Regions_204counties_change <- read.csv('D:/GBD/table4s/Global_21Regions_204counties_change.csv', header=T)

order_global_21regions_204countries <- read.csv('D:/GBD/table4s/order_global_21regions_204countries.csv', header=T)

```

```{R}

YLDs1990_number_226 <- Global_21Regions_204counties_1990to2021 %>%

select("location_name","year","sex_name","age_name","measure_name","metric_name","val","upper", "lower" ) %>%

filter(year == "1990",

sex_name == "Both",

age_name == "All ages",

measure_name == "YLDs (Years Lived with Disability)",

metric_name == "Number")

YLDs1990_number_226 <- YLDs1990_number_226 %>%

mutate(val = round(val,0),

upper = round(upper,0),

lower = round(lower,0),

YLDs_Number_1990 = paste0(val,' (',lower,',',upper,')'))

Order_factor <- factor(YLDs1990_number_226$"location_name", levels = order_global_21regions_204countries$"Country")

YLDs1990_number_226 <- YLDs1990_number_226[order(Order_factor), ]

```

```{R}

YLDs1990_ASR_226 <- Global_21Regions_204counties_1990to2021 %>%

select("location_name","year","sex_name","age_name","measure_name","metric_name","val","upper", "lower" ) %>%

filter(year == "1990",

sex_name == "Both",

age_name == "Age-standardized",

measure_name == "YLDs (Years Lived with Disability)",

metric_name == "Rate")

YLDs1990_ASR_226 <- YLDs1990_ASR_226 %>%

mutate(val = round(val,1),

upper = round(upper,1),

lower = round(lower,1),

YLDs_ASR_1990 = paste0(val,' (',lower,',',upper,')'))

Order_factor <- factor(YLDs1990_ASR_226$"location_name", levels = order_global_21regions_204countries$"Country")

YLDs1990_ASR_226 <- YLDs1990_ASR_226[order(Order_factor), ]

```

```{R}

YLDs2021_number_226 <- Global_21Regions_204counties_1990to2021 %>%

select("location_name","year","sex_name","age_name","measure_name","metric_name","val","upper", "lower" ) %>%

filter(year == "2021",

sex_name == "Both",

age_name == "All ages",

measure_name == "YLDs (Years Lived with Disability)",

metric_name == "Number")

YLDs2021_number_226 <- YLDs2021_number_226 %>%

mutate(val = round(val,0),

upper = round(upper,0),

lower = round(lower,0),

YLDs_Number_2021 = paste0(val,' (',lower,',',upper,')'))

Order_factor <- factor(YLDs2021_number_226$"location_name", levels = order_global_21regions_204countries$"Country")

YLDs2021_number_226 <- YLDs2021_number_226[order(Order_factor), ]

```

```{R}

YLDs2021_ASR_226 <- Global_21Regions_204counties_1990to2021 %>%

select("location_name","year","sex_name","age_name","measure_name","metric_name","val","upper", "lower" ) %>%

filter(year == "2021",

sex_name == "Both",

age_name == "Age-standardized",

measure_name == "YLDs (Years Lived with Disability)",

metric_name == "Rate")

YLDs2021_ASR_226 <- YLDs2021_ASR_226 %>%

mutate(val = round(val,1),

upper = round(upper,1),

lower = round(lower,1),

YLDs_ASR_2021 = paste0(val,' (',lower,',',upper,')'))

Order_factor <- factor(YLDs2021_ASR_226$"location_name", levels = order_global_21regions_204countries$"Country")

YLDs2021_ASR_226 <- YLDs2021_ASR_226[order(Order_factor), ]

```

```{R}

change_YLDs_ASR_226 <- Global_21Regions_204counties_change %>%

select("year_start","year_end","location_name","sex_name","age_name","measure_name","metric_name","val","upper", "lower" ) %>%

filter(year_start == "1990",

year_end == "2021",

sex_name == "Both",

age_name == "Age-standardized",

measure_name == "YLDs (Years Lived with Disability)",

metric_name == "Rate")

change_YLDs_ASR_226 <- change_YLDs_ASR_226 %>%

mutate(val = round(val*100,1),

upper = round(upper*100,1),

lower = round(lower*100,1),

YLDs_ASR_change = paste0(val,' (',lower,',',upper,')'))

Order_factor <- factor(change_YLDs_ASR_226$"location_name", levels = order_global_21regions_204countries$"Country")

change_YLDs_ASR_226<- change_YLDs_ASR_226[order(Order_factor), ]

```

```{R}

TableS4_YLDs <- data.frame(Location = YLDs1990_number_226$location_name,

"1990_No_(95%_UI)" = YLDs1990_number_226$YLDs_Number_1990,

"1990_ASRs_per_100000_(95%_UI)" = YLDs1990_ASR_226$YLDs_ASR_1990,

"2021_No_(95%_UI)" = YLDs2021_number_226$YLDs_Number_2021,

"2021_ASRs_per_100000_(95%_UI)" = YLDs2021_ASR_226$YLDs_ASR_2021,

"Percentage_change_in_the_ASRs_per_100000" = change_YLDs_ASR_226$YLDs_ASR_change)

```

```{R}

write.csv(TableS4_YLDs,file = "D:/GBD/table4s/TableS4_YLDs.csv")

```

2. R code for Fig 1:

install.packages("dplyr")

library(dplyr)

library(tidyverse)

library(readxl)

library(tidyr)

library(rnaturalearth)

library(rnaturalearthdata)

library(sf)

library(mapdata)

rm(list = ls())

df=read.csv("d:/GBD/地图/shijie.csv",header = T)

load("d:/GBD/地图/GBD.Rdata")

dfall = read.csv("d:/GBD/地图/shijie.csv", header = TRUE)

colnames(df)

labelx="Prevalence"

dfx = df %>%

dplyr::filter(measure_name == labelx) %>%

dplyr::filter(sex_name == "Both") %>%

dplyr::filter(age_id == 27) %>%

dplyr::filter(metric_name == "Rate")

print(dim(dfx))

dfplot= dfx %>% filter(location_id %in% namex$location_id ) %>%

select(location_id,val) %>% left_join(.,namex)

color1 <- c("#ed1c24",

"#f25a29",

"#f9971e",

"#fcd116",

"#ffe600",

"#d1c328",

"#8cbf4d",

"#4f9a85",

"#2b7bb8",

"#2468a2")

colorx1=color1

index="YLDs (Years Lived with Disability)"

ASR=dfplot %>% select(location_id,location,val) %>%

mutate(val=val/1)

df_asr=left_join(df_world,ASR)

xmin=min(na.omit(df_asr$val))

# Calculate the quantile breaks

breaks <- quantile(ASR$val, probs = seq(0, 1, length.out = 11), na.rm = TRUE)

breaks=breaks[-1]

print(breaks)

breaks100=1*breaks

formatted_breaks = vector("character", length(breaks100))

formatted_breaks[1] = paste(paste0(round(xmin,3)," -"), format(breaks100[1], digits = 4, nsmall = 1))

# Iterate over the breaks and format them

for (i in 2:length(breaks100)) {

formatted_breaks[i] = paste(paste0(format(breaks100[i - 1], digits = 4, nsmall = 1)), "-",paste0( format(breaks100[i], digits = 4, nsmall = 1)))

}

unb=unique(breaks)

if(unb[1]==xmin){

unb=unb[-1]

} else{

unb=unb

}

print(formatted_breaks)

x=cut(df_asr$val, breaks = c(xmin, unb))

print(na.omit(unique(x)))

xlen=length(na.omit(unique(x)))

xstar=length(formatted_breaks)-xlen+1

formatted_breaks=formatted_breaks[xstar:length(formatted_breaks)]

# Cut break for Prevalence

df_asr = df_asr %>%

mutate(asr = replace_na(val, -99)) %>%

mutate(asr_cut = cut(asr, breaks = c(xmin, unb),

labels = formatted_breaks)) %>%

filter(!is.na(asr_cut))

ggplot(df_asr %>% na.omit()) +

geom_sf(aes(geometry = geometry, fill = asr_cut),size = 0.1)+

#labs(title = paste0("Age-standardized ",index," Rate (Per 100,000),both sexes in 2017")) +

scale_fill_manual(#name="Anaemia prevalence",

values = rev(colorx1),

guide = guide_legend(reverse=T))+

guides(fill = guide_legend(ncol = 2, title = labelx))->p

p1=p+ theme(axis.text.x = element_blank(),

axis.ticks.x = element_blank(),

legend.position = c(0.13, 0.29),

legend.background = element_blank(),

legend.key = element_blank(),

legend.title=element_text(size=8),

legend.text=element_text(size=8),

panel.grid.major = element_blank(),

panel.grid.minor = element_blank(),

panel.border = element_blank(),

panel.background = element_blank())

p1

library(patchwork)

theme_map_sub <- theme_void() + labs(x="", y="") + theme_bw() +

#theme(plot.background = element_rect(fill = "lightgrey", colour = NA))+

theme(text = element_text(size = 12), # 小地图上方文字的大小

panel.grid.major = element_blank(),

panel.grid.minor = element_blank(),

legend.position = 'none',

axis.text = element_blank(),

axis.ticks = element_blank(),

plot.background = element_rect(fill = "transparent"),

plot.title = element_text(vjust = 0.01, hjust = 0.5))

#sub1 ###################### Caribbean and central America

x_location = c(-92,-59); y_location = c(7,28)

sub1 <- p1+

coord_sf(xlim = x_location, ylim = y_location, expand = FALSE) +

ggtitle('Caribbean and central America') +

theme(legend.position = "none")+

theme_map_sub

#sub2 ###################### Caribbean and central America

x_location = c(45,55.8)

y_location = c(21,31.5)

#######################

sub2 <- p1+

coord_sf(xlim = x_location, ylim = y_location, expand = FALSE) +

ggtitle('Persian Gulf') +

theme(legend.position = "none")+

theme_map_sub

#sub3 ###################### Caribbean and central America

x_location = c(12.5,32);y_location = c(34.5,50)

#######################

sub3 <- p1+

coord_sf(xlim = x_location, ylim = y_location, expand = FALSE) +

ggtitle('Balkan Peninsula')+

theme(legend.position = "none")+

theme_map_sub

#sub4 ###################### Caribbean and central America

x_location = c(94.9,119.1)

y_location = c(-9.2,9)

#######################

sub4 <- p1+

coord_sf(xlim = x_location, ylim = y_location, expand = FALSE) +

ggtitle('Southeast Asia')+

theme(legend.position = "none")+

theme_map_sub

#sub5 ###################### Caribbean and central America

x_location = c(-17.8,-7)

y_location = c(6.5,15.8)

#######################

sub5 <- p1+

coord_sf(xlim = x_location, ylim = y_location, expand = FALSE) +

ggtitle('West Africa')+

theme_map_sub+

theme(legend.position = "none",

plot.title = element_text(hjust = 0.5, size =10))

#sub6 ###################### Caribbean and central America

x_location = c(30.5,38.5)

y_location = c(28.4,35.9)

#######################

sub6 <- p1+

coord_sf(xlim = x_location, ylim = y_location, expand = FALSE) +

ggtitle('Eastern\n Mediterranean')+

theme_map_sub+

theme(legend.position = "none",

plot.title = element_text(hjust = 0.5, size =10))

#sub7 ###################### Caribbean and central America

x_location = c(4.7,27.5)

y_location = c(48,59)

#######################

sub7 <- p1+

coord_sf(xlim = x_location, ylim = y_location, expand = FALSE) +

ggtitle('Northern Europe')+

theme(legend.position = "none")+

theme_map_sub

plot1 <- (sub1 + sub2 + sub3 + sub4) + plot_layout(nrow = 1)

plot2 <- (sub5 | sub6) / sub7 + plot_layout(height = c(2.8, 2.5))

plot3 <- plot1 | plot2 + plot_layout(widths = c(1, 15))

px <- p1/ plot3 + plot_layout(height = c(2,1), widths = c(2,1.2))

ggsave("map-pro.jpeg",width = 12,height = 10)

3. R code for Fig 2A:

library(ggplot2)

library(reshape2)

library(dplyr)

library('readxl')

setwd("D:/GBD/fig1")

IBD_fig1<- read.csv('IBD_allfig1.csv',header = T)

str(IBD_fig1$metric_name)

table(IBD_fig1$metric_name)

table(IBD_fig1$measure_name)

unique(IBD_fig1$age_name)

age1 <- c("<5 years","5-9 years","10-14 years","15-19 years","20-24 years",

"25-29 years","30-34 years","35-39 years","40-44 years","45-49 years",

"50-54 years","55-59 years","60-64 years","65-69 years","70-74 years",

"75-79 years","80-84","85-89","90-94","95+ years")

data3<- subset(IBD_fig1,IBD_fig1$year==2021& #提取2021年

(IBD_fig1$age_name %in% age1 ) & #提取不同年龄段

IBD_fig1$sex_name!="Both"& #把男女分开

IBD_fig1$location_name=='Global'& #提取中国数据

IBD_fig1$metric_name== 'Number' & #提取10人数

IBD_fig1$measure_name=='Prevalence') #提取患病率数据

str(data3)

data3<-data3[,c("sex_name","age_name","val","upper","lower")]

data3$age_name<-gsub(" years","",data3$age_name)

data3$age_name <- factor(data3$age_name, levels = c("<5", "5-9", "10-14", "15-19", "20-24", "25-29", "30-34", "35-39", "40-44", "45-49", "50-54", "55-59", "60-64", "65-69", "70-74", "75-79", "80-84", "85-89", "90-94", "95+"))

data3 <- data3[order(data3$sex_name, data3$age_name),]

data3$val<-round(data3$val,0)

data3$Sex<-as.factor(data3$sex_name)

custom_colors <- c("Male" = "steelblue", "Female" = "#e31a1c")

library(ggplot2)

p3 <- ggplot(data3, aes(x = factor(age_name, levels = unique(age_name)),

y = ifelse(Sex == "Male", val, -val),

fill = Sex)) +

geom_bar(stat = 'identity') +

coord_flip() +

labs(x = 'Age', y = 'The Numbers of Prevalence') +

geom_text(aes(label = val,

hjust = ifelse(Sex == "Male", -0.4, 1.1)),

size = 2) +

scale_fill_manual(values = custom_colors) +

scale_y_continuous(labels = abs, expand = expansion(mult = c(0.2, 0.2))) +

theme_minimal() +

theme(panel.grid = element_blank(),

panel.background = element_blank(),

axis.line = element_blank())

print(p3)

data4<- subset(IBD_fig1,IBD_fig1$year==2021&

(IBD_fig1$age_name %in% age1 ) &

IBD_fig1$sex_name!="Both"&

IBD_fig1$location_name=='Global'&

IBD_fig1$metric_name== 'Number' &

IBD_fig1$measure_name=='Incidence')

data4<-data4[,c("sex_name","age_name","val","upper","lower")]

data4$age_name<-gsub(" years","",data4$age_name)

data4$age_name <- factor(data4$age_name, levels = c("<5", "5-9", "10-14", "15-19", "20-24", "25-29", "30-34", "35-39", "40-44", "45-49", "50-54", "55-59", "60-64", "65-69", "70-74", "75-79", "80-84", "85-89", "90-94", "95+"))

data4 <- data4[order(data4$sex_name, data4$age_name),]

data4$val<-round(data4$val,0)

data4$sex_name<-as.factor(data4$sex_name)

custom_colors <- c("Male" = "#1f78b4", "Female" = "#e31a1c")

data4$Sex<-data4$sex_name

library(ggplot2)

p4 <- ggplot(data4, aes(x = factor(age_name, levels = unique(age_name)),

y = ifelse(Sex == "Male", val, -val),

fill = Sex)) +

geom_bar(stat = 'identity') +

coord_flip() +

labs(x = 'Age', y = 'The Numbers of Incidence') +

geom_text(aes(label = val,

hjust = ifelse(Sex == "Male", -0.4, 1.1)),

size = 2) +

scale_fill_manual(values = custom_colors) +

scale_y_continuous(labels = abs, expand = expansion(mult = c(0.2, 0.2))) +

theme_minimal() +

theme(panel.grid = element_blank(),

panel.background = element_blank(),

axis.line = element_blank())

print(p4)

library(ggpubr)

ggarrange(p3,p4,ncol = 1)

4. R code for Fig 2b:

library(ggplot2)

library(reshape2)

library(dplyr)

library(readxl)

setwd("D:/GBD/fig1")

IBD_fig1 <- read.csv('IBD_allfig1.csv',header = T)

str(IBD_fig1$metric_name)

table(IBD_fig1$metric_name)

table(IBD_fig1$measure_name)

age1 <- c("<5 years","5-9 years","10-14 years","15-19 years","20-24 years",

"25-29 years","30-34 years","35-39 years","40-44 years","45-49 years",

"50-54 years","55-59 years","60-64 years","65-69 years","70-74 years",

"75-79 years","80-84","85-89","90-94","95+ years")

data1<- subset(IBD_fig1,IBD_fig1$year==2021&

(IBD_fig1$age_name %in% age1 ) &

IBD_fig1$sex_name!="Both"&

IBD_fig1$location_name=='Global'&

IBD_fig1$metric_name== 'Rate' &

IBD_fig1$measure_name=='YLDs (Years Lived with Disability)')

str(data1)

data1<-data1[,c("sex_name","age_name","val","upper","lower")]

data1$age_name<-gsub(" years","",data1$age_name)

data1$age_name <- factor(data1$age_name, levels = c("<5", "5-9", "10-14", "15-19", "20-24", "25-29", "30-34", "35-39", "40-44", "45-49", "50-54", "55-59", "60-64", "65-69", "70-74", "75-79", "80-84", "85-89", "90-94", "95+"))

data1 <- data1[order(data1$sex_name, data1$age_name),]

data1$val<-round(data1$val,2)

data1$Sex<-as.factor(data1$sex_name)

p3 <- ggplot(data = data1, aes(x = age_name, y = val, color = Sex, group = Sex)) +

geom_line() +

geom_point(size = 1) +

labs(x = 'Age', y = 'The Rate of YLDs') +

scale_fill_manual(values = c("#e31a1c", "steelblue")) +

scale_color_manual(values = c("#e31a1c", "steelblue")) +

geom_ribbon(aes(ymin = lower, ymax = upper, fill = Sex), alpha = 0.1, color = NA) +

scale_y_continuous(breaks = seq(0, 50, 10)) +

theme_minimal() +

theme(

panel.background = element_blank(),

panel.grid.major = element_blank(),

panel.grid.minor = element_blank(),

axis.line = element_line(color = "black"),

axis.text.x = element_text(angle = 45, hjust = 1)

)

print(p3)

data2<- subset(IBD_fig1,IBD_fig1$year==2021&

(IBD_fig1$age_name %in% age1 ) &

IBD_fig1$sex_name!="Both"&

IBD_fig1$location_name=='Global'&

IBD_fig1$metric_name== 'Rate' &

IBD_fig1$measure_name=='Incidence')

data2<-data2[,c("sex_name","age_name","val","upper","lower")]

data2$age_name<-gsub(" years","",data2$age_name)

data2$age_name <- factor(data2$age_name, levels = c("<5", "5-9", "10-14", "15-19", "20-24", "25-29", "30-34", "35-39", "40-44", "45-49", "50-54", "55-59", "60-64", "65-69", "70-74", "75-79", "80-84", "85-89", "90-94", "95+"))

data2 <- data2[order(data2$sex_name, data2$age_name),]

data2$val<-round(data2$val,2)

data2$Sex<-as.factor(data2$sex_name)

p4 <- ggplot(data = data2, aes(x = data2$age_name, y = data2$val, color = Sex, group = Sex)) +

geom_line() +

geom_point(size = 1.2) +

labs(x = 'Age', y = 'The Rate of Incidence') +

scale_fill_manual(values = c("#e31a1c","steelblue")) +

scale_color_manual(values = c("#e31a1c","steelblue")) +

geom_ribbon(aes(ymin = data2$lower, ymax = data2$upper, fill = Sex), alpha = 0.1, color = NA) +

scale_y_continuous(breaks = seq(0, 1800, 200)) +

theme_minimal() +

theme(

panel.background = element_blank(),

panel.grid.major = element_blank(),

panel.grid.minor = element_blank(),

axis.line = element_line(color = "black"),

axis.text.x = element_text(angle = 45, hjust = 1)

)

print(p4)

library(ggpubr)

ggarrange(p3,p4,ncol = 2)

ggarrange(p1,p3,p2,p4,ncol = 2,nrow=2)

5. R code for Fig 3:

setwd("D:/GBD/fig2")

library(ggplot2)

library(reshape2)

library(dplyr)

library('readxl')

IBD_SHUANGZHOU.csv <- read.csv('IBD_SHUANGZHOU.csv',header = T)

unique(IBD_SHUANGZHOU.csv$age_name)

data5<- subset(IBD_SHUANGZHOU.csv,

IBD_SHUANGZHOU.csv$sex_name!="Both"&

IBD_SHUANGZHOU.csv$location_name=='Global'&

(IBD_SHUANGZHOU.csv$metric_name %in% c('Number','Rate') ) &

IBD_SHUANGZHOU.csv$measure_name =='Prevalence')

unique(data5$metric_name)

str(data5)

dataxuan <- data5[,c("sex_name","age_name","metric_name","year","val","upper","lower")]

p5 <- ggplot() +

geom_bar(data = subset(dataxuan, age_name =="All ages"),

mapping=aes(x = year, y = val, fill = sex_name),

stat = "identity",

position = position_dodge(width = 0.8),

width = 0.7) +

geom_errorbar(data = subset(dataxuan, age_name =="All ages"),

mapping=aes(x = year, ymin = lower, ymax = upper,

group = sex_name ),

position = position_dodge(width = 0.8),

width = 0.25,

color = "black") +

geom_line(data = subset(dataxuan, age_name =="Age-standardized"),

aes(x = year, y = val*40000, color = sex_name),

size = 1) +

geom_ribbon(data = subset(dataxuan, age_name =="Age-standardized"),

aes(x = year, ymin = lower*40000, ymax = upper*40000,

fill = sex_name),

alpha = 0.2) +

scale_y_continuous(

name = "Prevalence Number",

sec.axis = sec_axis(~ . * 0.000025, name = "Age-standardized Prevalence rate per 100,000")

) +

scale_fill_manual(values = c("#e31a1c", "steelblue","blue", "red"), name = "Number") +

scale_color_manual(values = c("#e31a1c", "steelblue"), name = "Rate") +

labs(x = "Year") +

theme_minimal() +

theme(

panel.grid.major = element_blank(),

panel.grid.minor = element_blank()

)

print(p5)

data6<- subset(IBD_SHUANGZHOU.csv,

IBD_SHUANGZHOU.csv$sex_name!="Both"&

IBD_SHUANGZHOU.csv$location_name=='Global'&

(IBD_SHUANGZHOU.csv$metric_name %in% c('Number','Rate') ) &

IBD_SHUANGZHOU.csv$measure_name =='YLDs (Years Lived with Disability)')

datashuangzhouincident <- data6[,c("sex_name","age_name","metric_name","year","val","upper","lower")]

p6 <- ggplot() +

geom_bar(data = subset(datashuangzhouincident, age_name =="All ages"),

mapping=aes(x = year, y = val, fill = sex_name),

stat = "identity",

position = position_dodge(width = 0.8),

width = 0.7) +

geom_errorbar(data = subset(datashuangzhouincident, age_name =="All ages"),

mapping=aes(x = year, ymin = lower, ymax = upper,

group = sex_name ),

position = position_dodge(width = 0.8),

width = 0.25,

color = "black") +

geom_line(data = subset(datashuangzhouincident, age_name =="Age-standardized"),

aes(x = year, y = val*40000, color = sex_name),

size = 1) +

geom_ribbon(data = subset(datashuangzhouincident, age_name =="Age-standardized"),

aes(x = year, ymin = lower*40000, ymax = upper*40000,

fill = sex_name),

alpha = 0.2) +

scale_y_continuous(

name = "YLDs Number",

sec.axis = sec_axis(~ . * 0.000025, name = "Age-standardized YLDs rate per 100,000")

) +

scale_fill_manual(values = c("#e31a1c", "steelblue","blue", "red"), name = "Number") +

scale_color_manual(values = c("#e31a1c", "steelblue"), name = "Rate") +

labs(x = "Year") +

theme_minimal() +

theme(

panel.grid.major = element_blank(),

panel.grid.minor = element_blank()

)

print(p6)

5. R code for Fig 4:

library(dplyr)

library(ggplot2)

file_path <- "D:/GBD/7.FigureS11 /shaixuan.csv"

data <- read.csv(file_path, stringsAsFactors = FALSE)

df_incidence <- data %>%

filter(measure_name == "Incidence")

df_incidence$location_name <- factor(df_incidence$location_name,

levels = rev(unique(data$location_name)))

agg <- df_incidence %>%

group_by(cause_name) %>%

summarise(total_val = sum(val)) %>%

arrange(total_val)

df_incidence$cause_name <- factor(df_incidence$cause_name, levels = agg$cause_name)

custom_colors <- rev(c(

"#E41A1C",

"#377EB8",

"#4DAF4A",

"#984EA3",

"#FF7F00",

"#FFFF33",

"#A65628",

"#F781BF",

"#999999",

"#66C2A5",

"#FC8D62",

"#8DA0CB",

"#E78AC3",

"#A6D854",

"#FFD92F",

"#E5C494"

))

ordered_causes <- levels(df_incidence$cause_name)

names(custom_colors) <- ordered_causes

df_incidence <- df_incidence %>%

arrange(val)

custom_colors <- custom_colors[rev(names(custom_colors))]

ggplot(df_incidence, aes(y = location_name, x = val, fill = cause_name)) +

geom_bar(stat = "identity") +

labs(

x = "Incidence rate (per 100000 population)",

y = "GBD regions ",

fill = "Cause of forearm fractures"

) +

scale_fill_manual(values = custom_colors) +

guides(fill = guide_legend(reverse = TRUE)) +

theme_minimal() +

theme(

panel.grid = element_blank(),

axis.line.x = element_line(color = "black"),

axis.ticks.x = element_line(color = "black")

) +

scale_x_continuous(breaks = scales::pretty_breaks(n = 5))
